# Understanding the Role of the Gut Microbiome in Brain Development and Its Association With Neurodevelopmental Psychiatric Disorders

**DOI:** 10.3389/fcell.2022.880544

**Published:** 2022-04-14

**Authors:** Somarani Dash, Yasir Ahmed Syed, Mojibur R. Khan

**Affiliations:** ^1^ Life Sciences Division, Institute of Advanced Study in Science and Technology (IASST), Guwahati, India; ^2^ Academy of Scientific and Innovative Research (AcSIR), Ghaziabad, India; ^3^ School of Biosciences and Neuroscience and Mental Health Research Institute, Cardiff University, Hadyn Ellis Building, Cardiff, United Kingdom

**Keywords:** gut microbiome, inflammation, autism spectrum disorder, attention-deficit/hyperactivity disorder, schizophrenia, neurodevelopmental psychiatric disorder, drug resistance, probiotics

## Abstract

The gut microbiome has a tremendous influence on human physiology, including the nervous system. During fetal development, the initial colonization of the microbiome coincides with the development of the nervous system in a timely, coordinated manner. Emerging studies suggest an active involvement of the microbiome and its metabolic by-products in regulating early brain development. However, any disruption during this early developmental process can negatively impact brain functionality, leading to a range of neurodevelopment and neuropsychiatric disorders (NPD). In this review, we summarize recent evidence as to how the gut microbiome can influence the process of early human brain development and its association with major neurodevelopmental psychiatric disorders such as autism spectrum disorders, attention-deficit hyperactivity disorder, and schizophrenia. Further, we discuss how gut microbiome alterations can also play a role in inducing drug resistance in the affected individuals. We propose a model that establishes a direct link of microbiome dysbiosis with the exacerbated inflammatory state, leading to functional brain deficits associated with NPD. Based on the existing research, we discuss a framework whereby early diet intervention can boost mental wellness in the affected subjects and call for further research for a better understanding of mechanisms that govern the gut-brain axis may lead to novel approaches to the study of the pathophysiology and treatment of neuropsychiatric disorders.

## 1 Introduction

The gut microbiota with trillions of microbial cells and thousands of species profoundly influences human physiology, including various diseases and disorders. The human gut microbiota starts colonizing during the pregnancy period (Collado et al., 2016). Collectively the genome of all these microorganisms represents the microbiome. The process of development and colonization of gut microbes co-occurs with brain development during pregnancy in a coordinated way until the first few years after birth (Jena et al., 2020; Acuña et al., 2021). An imbalance in the gut microbiome during the critical developmental phase can influence the overall developmental process, primarily neuronal plus glial development and maturation (Borre et al., 2014; Marín, 2016). The microbiota composition exhibits the highest intra- and inter-individual variability during the first 12 months of post-natal development until it reaches a stable adult-like organization at the age of ∼ 3 years and can influence the process associated with brain development and in shaping the immune profile of the individual. (Koenig et al., 2011; Bäckhed et al., 2015; Gensollen et al., 2016). Early-life colonization of the host’s mucosal surfaces is crucial for the development and maturation of the host’s immune system in a healthy individual (Gensollen et al., 2016).

**FIGURE 1:**
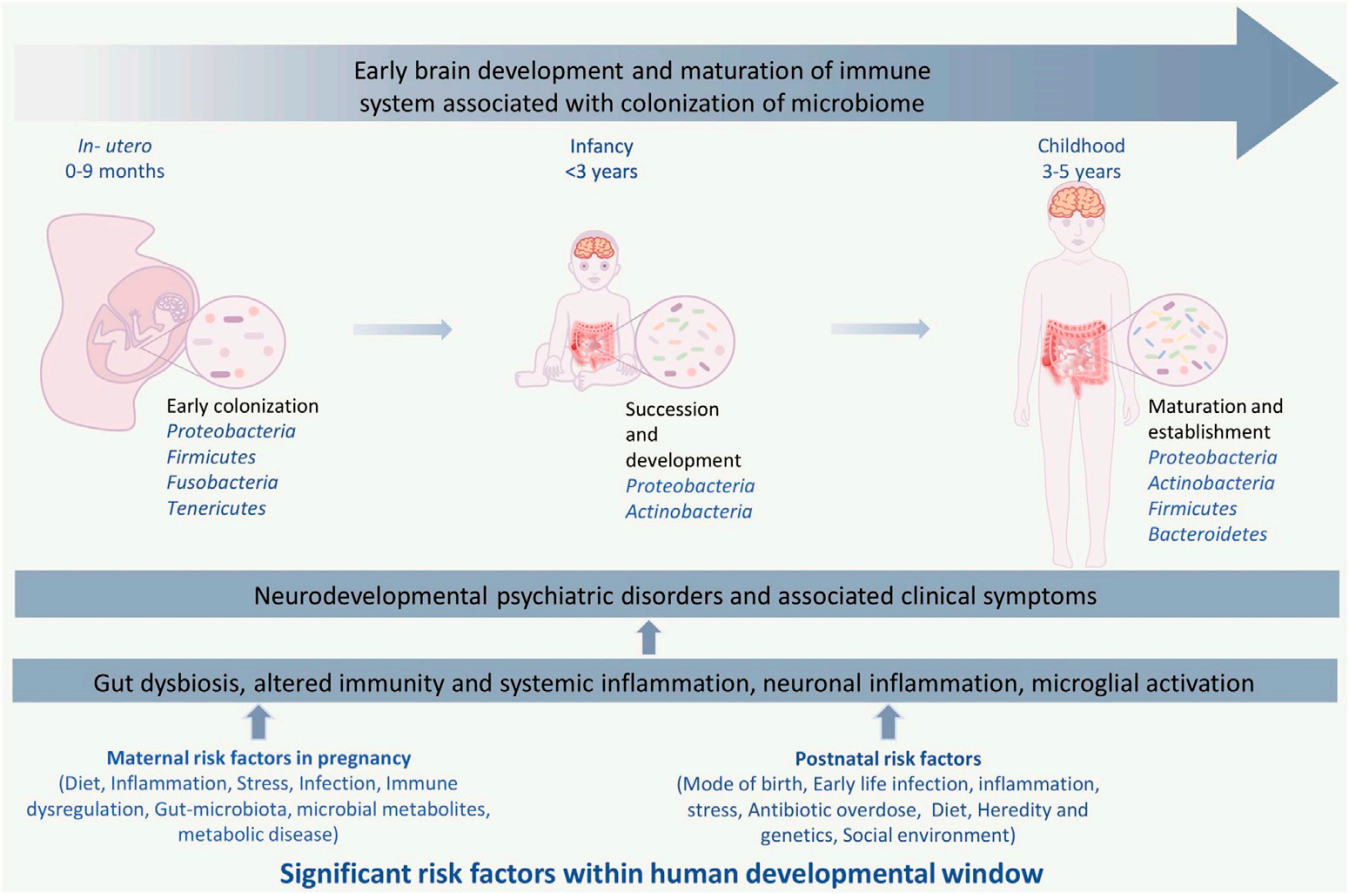
Significant risk factors affecting events with in human developmental window could bring on neurodevelopmental psychiatric disorders. The critical developmental window spans from the fetal stage until childhood, which consists of early colonization and development of the microbiome, development of the brain and nervous system, and development and maturation of the immune system. Within this period, the composition and diversity of the gut microbiome, genetics, maternal, and other reverent factors can alter the overall developmental homeostasis by causing disturbances in the immune system. Gut dysbiosis, immune alteration, and other factors induce microglial activation *via* inflammatory response, which leads to systemic and neuroinflammation. This negatively affects the brain development process and leads to abnormal brain development and functionality, including anxiety, depression, intellectual disability, and behavioral abnormality that can be seen in neurodevelopmental and psychotic disorders.

However, exposure to factors such as maternal immune activation (MIA), poor diet, disease/infections and antibiotic overdose can lead to early life gut dysbiosis (Vangay et al., 2015; Neuman et al., 2018; Li et al., 2021). The altered gut microbiome can cause dysregulated immune activation, igniting systemic inflammation resulting in atypical brain development leading to symptoms associated with neurodevelopmental psychiatric disorders (NPD) (Figure 1) (Garay and McAllister, 2010; Han et al., 2021; J.; Lu and Claud, 2019; Munawar et al., 2021). Hence, the presence of a balanced microbiome is needed for the proper functioning of the immune system, which in turn regulates the neurodevelopmental trajectories (Garay and McAllister, 2010; Gensollen et al., 2016; Sotgiu et al., 2020).

NPDs are a spectrum of disorders arising from atypical brain development resulting in cognitive, emotional, and motor deficits (Kapoor, 2002; Villagomez et al., 2019; Munawar et al., 2021). These include attention deficit hyperactivity disorders (ADHD), autism spectrum disorders (ASD), and schizophrenia (SCZ) which affect children in their daily lives through the early adolescence and later periods of life. These NPDs are also associated with psychotic symptoms and behavioral abnormalities characterized by positive symptoms such as psychotic hallucinations, the eccentric overflow of thoughts, and negative symptoms like insensibility, emotional and interactive withdrawal, lethargy, etc. (Cowen et al., 2012). These behavioral abnormalities often change or maturate and become more severe as a child grows older; some disabilities and behavioral abnormalities remain permanent (Klimkeit et al., 2016).

**FIGURE 2:**
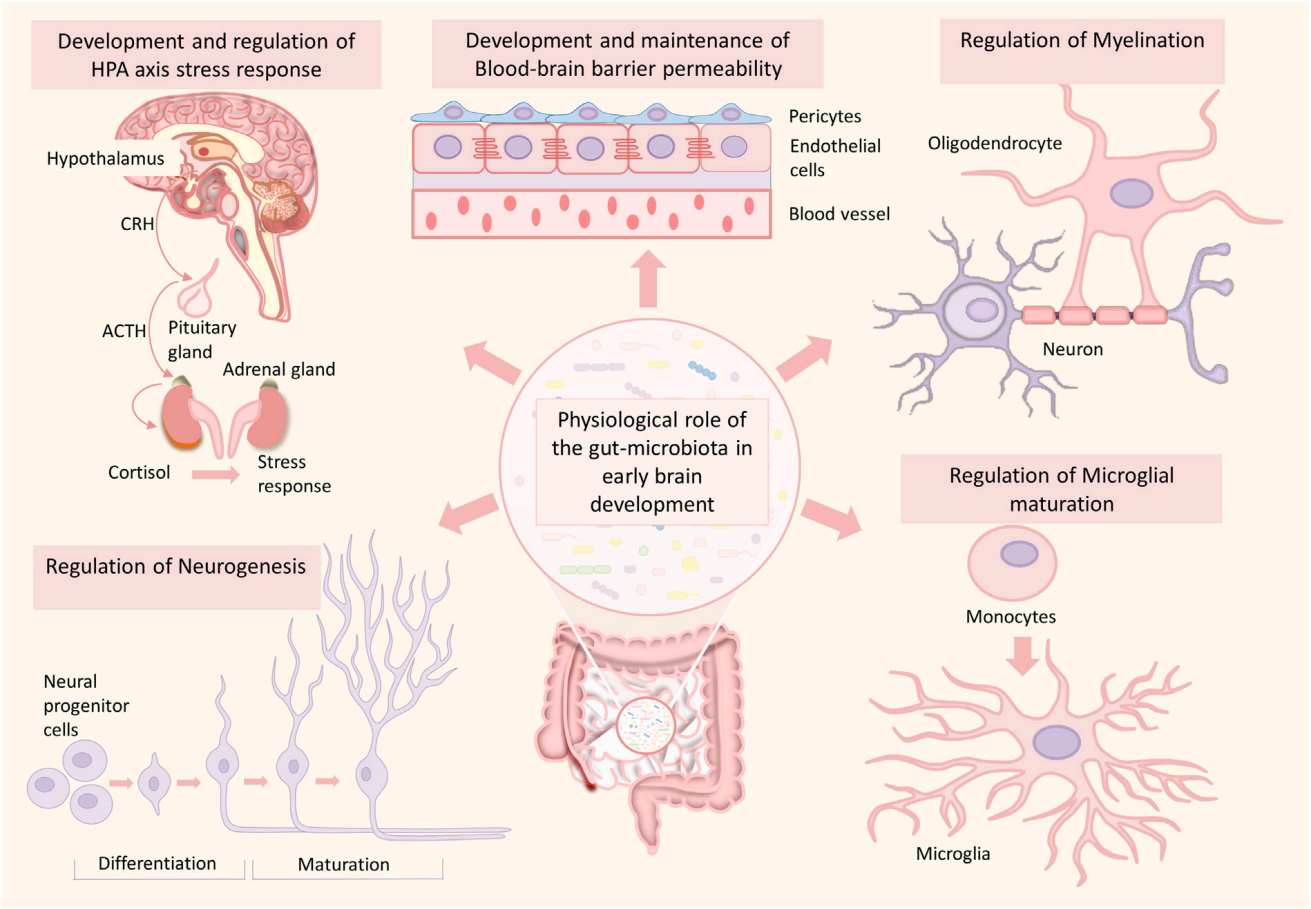
Role of the gut microbiome in healthy brain development. The gut microbiome plays an essential role in various processes of brain development such as neurogenesis, myelination, microglial maturation, development and maintenance of blood-brain barrier integrity, development of HPA-axis, and HPA-axis stress response. Any alterations in this developmental process can significantly increase the risk for neurodevelopmental disorders.

An increasing number of preclinical and clinical studies have been focusing on how the interaction between the complex gut-microbial ecosystem and central nervous system can regulate brain development and its association with NPDs (Figure 2) (Yap and Martin, 2015; Ernesto Martínez-González and Andreo-Martínez, 2019; Bull-Larsen and Hasan, 2019; Yuan et al., 2019; Checa-Ros et al., 2021; Kelly et al., 2021). However, most of these studies have explored the relationship on core pathology associated with one clinical subtype of NPDs. It is evident that there is a need for a greater understanding of the complex coordinated underlying pathways that’s span major NPDs. In this timely review, we discuss the recent evidence establishing the direct role of commensal gut microbes and associated metabolites on human brain development and how alterations in gut microbiome brain axis mediated communications play a role in the pathology of ASD, ADHD, and SCZ, which stems from aberrant brain development. Secondly, we propose a hypothetical framework illustrating an association between gut microbiota and inflammation affecting brain development and preventive measures in association with diet and probiotic therapy at crucial time points to mitigate the initiation and proper management of existing neurodevelopmental psychiatric disorders.

## 2 Development of Gut Microbiota

Following birth, the colonization of microbes initiates and is very much influenced by the mode of delivery (normal delivery or C-section) (Biasucci et al., 2010; Wampach et al., 2017). Some studies have shown that microbial colonization begins *in-utero* with the discrete microbial community present in the umbilical cord blood, placenta, and amniotic fluid and may carry out their role in the foetal developmental process (Aagaard et al., 2014; Collado et al., 2016). Although some studies have defined the microbial composition of the fetal meconium has been defined in several studies (Moles et al., 2013; Stinson et al., 2019), others have linked premature birth with the decreased microbial diversity within the meconium microbiome (Ardissone et al., 2014). Interestingly some recent studies suggest a similarity within the microbial profile in between placenta, fetal meconium, and amniotic fluid (He et al., 2020; Senn et al., 2020). Younge et al. have confirmed maternal-fetalin utero-translocation of the gut microbiota using a mouse model (Younge et al., 2019). The preterm neonate meconium has been shown to have abundant microbes belonging to *Lactobacillus, Staphylococcus*, and *Enterobacteriales* (Madan et al., 2012). With low diversity and high inter-individual variability, the meconium microbiome is more abundant with genera *Bacillus, Escherichia/Shigella*, and *Enterococcus*, whereas *Bacteroides* and *Bifidobacterium* are less abundant concerning the fecal microbiome of infants (Moles et al., 2013; Bäckhed et al., 2015). The placenta is most abundantly colonized by phyla *Proteobacteria* and *Bacteroidetes* and various other phyla like *Firmicutes, Fusobacteria, Tenericutes*, and genera *Mycoplasma and Ureaplasma* (Brotman, 2011; Aagaard et al., 2014). At the time of birth, the babies born through standard delivery through the vagina are colonized mainly by the maternal vaginal microbial population, predominantly *Lactobacillus* and *Prevotella* (Dominguez-Bello et al., 2010). In contrast, cesarean section delivered babies are more exposed and colonized by *Staphylococcus* and *Corynebacterium* closer to the skin microbiome (Dominguez-Bello et al., 2010). The typical microbiome composition of a newborn is mainly dominated by *Proteobacteria* and *Actinobacteria*, where the former dominates immediately after birth and later towards the 4 months of age (Bäckhed et al., 2015; Dogra et al., 2015). During the 1st year of life, the gut of the new-born infant is predominantly inhabited by *Bifidobacterium, Enterococcus, Escherichia/Shigella, Streptococcus*, *Bacteroides,* and *Rothia* gut-microbial population closer to maternal microbiota in addition to *Clostridium, Ruminococcus, Veilonella, Roseburia, Akkermansia, Alistipes, Eubacterium, Faecalibacterium, and Prevotella,* species. Moreover, variation in the maternal microbiota has also been suggested as modulating factor of microbiome composition in offspring (Dotterud et al., 2015). The 1st 3 years of the lifetime are more crucial for establishing a healthy and stable structured microbiome. It has been reported that the process of the microbiome and neuronal development coincides in an intense and coordinated way within this critical time frame and is most vulnerable to disruption (Sharon et al., 2016; Jena et al., 2020; Acuña et al., 2021). A gut dysbiosis at such period has been reported to bring about many NDD/NPD like ADHD, ASD, SCZ, intellectual as well as learning disability, and behavioral problems (Borre et al., 2014; Chrobak et al., 2016; Bojović et al., 2020). Moreover, after this period, microbiota reconstitution does not normalize the behavioral phenotype or neurochemical disturbances during the critical developmental period (Sudo et al., 2004; Heijtz et al., 2011; Click or tap here to enter text; Clarke et al., 2013). Hence, maintaining a healthy and well-structured microbiota is essential during prenatal and postnatal periods up to a particular developmental phase.

## 3 Role of Gut Microbes in Early Brain Development

Emerging studies provide evidence about the active role of microbiota during central nervous system development. It is now clear that gut microbes play an active role in the neurodevelopmental processes (Figure 2), including the establishment of the blood-brain barrier (BBB) (Parker et al., 2020), neurogenesis (Cerdó et al., 2020), maturation of microglia (Erny et al., 2015), and myelination (Hoban et al., 2016; Duncan and Watters, 2019). These processes are critical in shaping animal behavior and cognition. Various dietary components released from the gut are needed for the developing brain for neuronal cell maturity and proper functions (Prado and Dewey, 2014; Ratsika et al., 2021). Also, recent evidence describes that gut microbes can directly facilitate the development processes in the brain, which have long-lasting consequences in health (J. Lu and Claud, 2019).

### 3.1 Blood-Brain Barrier

The BBB establishes early *in-utero*, which constitutes capillary endothelial cells sealed by tight junction proteins, pericytes, and astrocytes, forming the restrictive barrier between the brain and systemic circulation. It also facilitates the exchange of molecules and nutrients for proper maintenance and functioning of the brain (Obermeier et al., 2013; Braniste et al., 2014). The presence of balanced gut microbiota and microbial-derived metabolites such as SCFAs are essential in regulating the formation and maintenance of intact BBB (Obermeier et al., 2013; Braniste et al., 2014; Michel and Prat, 2016). The permeability of BBB in developing sterile fetuses decreases towards adulthood (Mollgaard and Saunders, 1986). In germ-free (GF) mice, the permeability of BBB increases for macro-molecules due to reduced expression of basic junctional proteins occludin and claudin-5 in the brain endothelial layer (Braniste et al., 2014). Microbial colonization of the gut or administration of butyrate, a short-chain fatty acid (SCFA) produced by gut-microbial fermentation, has been shown to reduce the blood-brain permeability in GF mice (Braniste et al., 2014).

### 3.2 Neurogenesis

Neurogenesis refers to the development of new functional neurons through differentiation of the neural stem/progenitors’ cells (Gage, 2019; Cosacak et al., 2020). The functionality of neurogenesis and neuronal plasticity is critical for learning, memory, cognition, and stress response, especially in the hippocampus, as the cognitive center (Kempermann, 2019). A balanced gut-microbiota is involved directly or indirectly in maintaining the microenvironment to support the process of neuronal development (Sarubbo et al., 2022). A recent comparative study between GF and SPF mice outlines a range of gut microbial metabolites that can cross through the placenta into the foetal compartment, with the ability to induce and regulate the prenatal developmental process (Pessa-Morikawa et al., 2022). In addition, PG, a bacterial cell wall component, gets crossed through the placenta to reach the foetal brain, there it activates Toll-like receptor 2 (TLR2), triggering an increase in FOXG1 expression, a crucial transcription factor in regulating the development and neurogenesis, thereby inducing neuronal proliferation in the forebrain area (Kaul et al., 2012; Humann et al., 2016). A recent study provides direct evidence linking modulation of microbial function and associated cytokine by microbiota to influence the process of neurogenesis (Salvo et al., 2020).

Further, the gut-microbes may indirectly affect neuronal-plasticity by regulating neuronal migration and maturation in CNS may be *via* regulation of ephrin B and reelin pathway, where ephrin B plays a pivotal role in the maintenance of the gut epithelial barrier integrity and reelin, a membrane glycoprotein responsible for neuronal migration (Allam-Ndoul et al., 2020; Grandi et al., 2019; D’Arcangelo, 2014; White and Getsios, 2014; Sentürk et al., 2011; Hafner et al., 2005).

Accumulating evidence suggests that gut microbes can impact the neural stem cells’ fate by coordinating with intricate pathways of differentiation and survival through neurotrophins and neurotransmitters in different areas of the brain (Sharon et al., 2016). The process of synapse development and maturation is associated with neuronal maturation and plasticity. Experimental administration of neonatal prebiotic (BGOs) in comparison to other prebiotic in 22 days old rats has been reported to elevate hippocampal expression of synaptophysin and brain-derived neurotrophic factor (BDNF). Synaptophysin, a synaptic vesicle protein, which controls the synaptic vesicle endocytosis kinetics, and BDNF, a nerve growth factor secreted by neurons, which also act as a signaling molecule for neuronal survival, growth, maturation maintenance of various brain cell populations, as well as the establishment of neuronal circuitry through the formation of the synapse (S. Williams et al., 2016).

Serotonin, a neurotransmitter and signaling molecule, can also be synthesized and released by gut microbes into the gut lumen, known to promote adult neurogenesis (Alenina and Klempin 2015). In addition, gut microbes have been reported to have an essential involvement in serotonergic signaling pathways in the gut and multiple regions of the brain (O’Mahony et al., 2015). Several studies have also reported the role of gut microbiota in regulating adult neurogenesis. By labeling proliferating cells with bromo-deoxyuridine GF mice brain, an increase in the adult dorsal hippocampal neurogenesis was reported compared to conventionally grown mice, and even upon microbial colonization, the phenotypical condition couldn’t be reversed. This indicates that the absence of microbes induces a dysregulated increase in adult dorsal hippocampal neurogenesis, and the microbial signals during crucial early life developmental window act as a controlling force in regulating neurogenesis in the hippocampus (Ogbonnaya et al., 2015).

Furthermore, the usage of antibiotics, which has a negative impact on gut microbiota, was associated with decreased neurogenesis (Möhle et al., 2016). The molecular mechanism that regulates the process of adult neurogenesis *via* microbiota and its associated metabolites is not very clear. It is suggested that neuroinflammatory mechanisms mediate this process along with humoral and metabolic pathways (Liu et al., 2022). A number of recent studies provide evidence to support the role of neuroinflammatory mechanisms. Intestinal bacteria maintain the enteric nervous system in adult mice through Toll-like receptor 2-induced neurogenesis (Yarandi et al., 2020).

Additionally, a decrease in BDNF mRNA level in the whole hippocampus was reported, particularly with a significant decline in neuronal proliferation and survivability in vagotomized mice (O’Leary et al., 2018). Furthermore, microbiome also indirectly influences hippocampal neurogenesis *via* regulating the neuronal immune system. Induction of acute colonic inflammation *via* administrating dextran sodium sulfate in mice reported a dysbiotic gut microbial composition, the increased hippocampal expression level of pattern recognition receptor and T-helper 17 cell-associated cytokines and ionized calcium-binding adapter molecule 1, a microglial activation marker, concomitant to depreciated adult hippocampal neurogenesis with consequent behavioral deficits (Salvo et al., 2020).

Early life stress, like a lack of social interaction, can also alter the stability of the gut microbiome (Cong et al., 2015), along with reduced neurogenesis and IL-6 and IL-10 level in the hippocampus of socially isolated mice as compared to grouped controls (Dunphy-Doherty et al., 2018). A reduction in hippocampal neurogenesis is strongly associated with impaired learning, anxiety, depressive-like behaviors, neuroinflammation, which again have an explicit association with structural alterations gut microbiome (Liu et al., 2022).

### 3.3 Myelination

A healthy/intact gut microbiome has been reported to modulate myelination (Hoban et al., 2016; Jena et al., 2020). Humans are born primarily with unmyelinated axons in the central nervous system (CNS) at the time of birth. Rapid myelination of maturing axons occurs within just a few years after childbirth by oligodendrocytes through the process of engagement and ensheathment (Lu et al., 2002; Williamson and Lyons, 2018), with a variable rate of myelination and myelin content over time (Benes, 1989), until early adulthood. (Lebel et al., 2012; Williamson and Lyons, 2018). Any aberration in this process may lead to long-lasting defects. Myelination has the most crucial role in cognitive function, and the scale of myelination has been linked with neuronal plasticity and function (Almeida and Lyons, 2017; Kaller et al., 2017). The gut microbiota regulates the critical process of myelination by regulating myelination-related gene expression in oligodendrocytes. Myelin deformities can have a detrimental impact on brain function and behavior (Gacias et al., 2016; Hoban et al., 2016; Ntranos and Casaccia, 2018). Most notably, the prefrontal cortex (PFC) area of the brain exhibits myelination at a later period, during the initial phase of an infantile life, which makes it more vulnerable to external influencing factors, like intestinal dysbiosis (Hoban et al., 2016). As in the case of GF mice, irregulated myelin formation in the PFC region has a harmful effect on social behavior (Gacias et al., 2016; Hoban et al., 2016). Furthermore, bacterial metabolites such as SCFAs have been demonstrated to have a beneficial impact over stress-induced behavioral difficulties, intestinal barrier dysfunctionalities, and in the regulation of the myelination process (Gacias et al., 2016; van de Wouw et al., 2018). SCFA butyrate, when orally administered, caused recovery of myelination impairments, intestinal physiology, and behavioral deficit in antibiotic-treated mice, indicating a pivotal role of gut microbiota in developing the microbiome-gut-brain (MGB) axis through regulation of myelination process in the PFC region (Keogh et al., 2021). Thus, the microbiota is crucial for myelination and maintenance of the plasticity of the myelin sheath.

### 3.4 Hypothalamus-Pituitary-Adrenal Axis

The endocrine-neurocrine interaction between the hypothalamus, pituitary gland, and adrenal gland in response to stress is known as the hypothalamus-pituitary-adrenal axis, where the corticotropin-releasing factor (CRF) plays the central role in the stress response *via* initiating a course of events that leads to the release of glucocorticoids from the adrenal cortex by regulating the HPA-axis (S. M. Smith and Vale, 2006). In developing the HPA-axis, commensal microbiota also plays a significant role (Sudo, 2012). In GF mice, dopamine deficiency, particularly in the frontal cortex hippocampus and striatum, is one of the major causes of improper stress regulation and anxiety-like behavior (Zarrindast and Khakpai, 2015). The administration of probiotic formulation consisting of probiotic strains *Lactobacillus helveticus* and *Bifidobacterium longum,* the level of anxiety decreased substantially (Partrick et al., 2021). Also, the comparison between GF and specific-pathogen-free (SPF) mice revealed increased CRF mRNA level in the hypothalamus of the GF mice, indicating an enhanced stress response related to HPA-axis (Sudo et al., 2004).

### 3.5 Microglial Development and Physiology

Microglia are resident immune cells (macrophages) that belong to the glial system. They account for 10–15% of the whole total glial cell count in the CNS (Hugh Perry, 1998), thoroughly distributed across the brain and the spinal cord (Lawson et al., 1990). Unlike the neuronal cells, microglial cells that constitute the innate immune system of CNS are derived from a subset of primitive macrophages that originates from the progenitor cell of the yolk sac (Ginhoux et al., 2013). The functionalities of microglia include immune defense and maintenance of the CNS. The microglial cells consistently survey their local microenvironment to detect pathogenic invasion or tissue damage throughout the CNS, guarded by the BBB (Daneman, 2012). Microglia also regulate neuronal proliferation, differentiation, and formation of synaptic connections (Graeber, 2010; Hughes, 2012), that helps in the refurbishing of post-natal neural circuits *via* controlling synaptic truncation during postnatal development of the mice (Tremblay et al., 2010; Paolicelli et al., 2011). Microglia contributes to both innate and adaptive immune system defense in the CNS. Abnormal activation of microglia aberrantly induces inflammation, which has been observed in most brain-related pathologies. Emerging evidence also suggests microglia directly affect neuronal pathology and contributes to disease progression (Perry et al., 2010; Helmut et al., 2011; Kingwell, 2012). Recent research data have demonstrated that microbiota has a vital role in the development and maturation of microglia (Erny et al., 2015; Abdel-Haq et al., 2019). However, upon damage or depletion of microglial macrophages and the yolk progenitor cells the bone marrow-derived macrophages can replenish them by developing and differentiation with the help of microbial signals at an early point (Erny et al., 2015; J.; Han et al., 2019). Moreover, in GF mice, the microglial cells show a significantly altered developmental state, with morphological characteristics and gene expression profile of a developmental and maturation arrest condition. These GF mice-derived microglia usually displayed a limited response towards viral infections and microbe-associated molecular patterns (MAMP), which interestingly rescued by SCFA administration (Hanamsagar et al., 2017).

## 4 Altered Gut Microbiota, Inflammation, and Neurodevelopmental Disorders

### 4.1 Altered Microbiome and Inflammation

In response to eliminating invading microorganisms such as bacteria, viruses, and other pathogens, the innate immune system triggers an intracellular signaling pathway *via* activation of toll-like receptors (TLRs) for gene expression of interferons, cytokines, and other immunological mediators (El-Zayat et al., 2019). The molecules during pathogenic elimination can also contribute to the development of inflammation response and so-called inflammatory mediators. Inflammation can also be triggered in response to endogenous factors such as predisposition to genetic abnormalities, behavioral anomalies of immune cells (autoimmunity/allergy), as well as external factors such as trauma, heat and cold stress, environmental toxicants, pathogens, diseases, etc. (Chen et al., 2018). Among pro-inflammatory mediator IL-1α, IL-1β, IL-2, IL-6, IL-8, IL-12, TNF-α, INF-γ and among anti-inflammatory mediator IL-4, IL-5, IL-10, TGF-β, C-reactive protein (CRP), and serum amyloid-A are considered as inflammation biomarkers (Brenner et al., 2014; Sands, 2015; Kany et al., 2019; Morris et al., 2020).

The gut microbes also have an essential role in the induction of inflammation. More specifically, the pathogenic/opportunistic pathogenic strains such *as E. coli* and *B. fragilis* from the *Enterobacteriace* family contribute to pathogenesis when outnumbered and eventually to inflammation (Hiippala et al., 2020). Lipopolysaccharides (LPS), a specific cell membrane component present mostly in Gram-negative bacteria like *E. coli* and *B fragilis* is endotoxic and, upon interaction with macrophages, cause the release of pro-inflammatory cytokine TNF-α, IL-6, IL-1, which lead to endocytic septic shock and often have fatal outcomes (Agarwal et al., 1995). The LPS from *Bacteroides* is also a potential stimulator of the innate immune system (Alexander and Rietschel, 2001). In systemic inflammation, there is a reported elevation in the expression of pro-inflammatory cytokines IL-6 and IL-8, which have been correlated with microbial taxa belonging to phylum *Proteobacteria* within total fecal microbiota (Biagi et al., 2010).

Moreover, the combination of different taxa of microbes can enhance the pathogenic effects that lead to inflammation. For example, co-infection of *E. coli* and *B. fragilis* can induce an increase in expression of TNF-α, keratinocytes-derived chemokine mRNA (KC mRNA), and proteins in peritoneal tissue and cause early peritonitis, abscess development, and death in the experimental mice model, which is not observed when infected singularly (J. M. Kim et al., 2000). Colonization of *Chloristidium difficle* has been reported to have an association with frequent occurrence of eczema, sensitization to allergy, and ectopic dermatitis (Lee et al., 2017).

In contrast to inflammation, inducing microbes, a fraction of gut microbiota can counteract the inflammation. These microbes can act either by directly inhibiting inflammation-inducing microbes, strengthening the gut epithelial/mucosal barrier or directly interacting with inflammation-inducing components of the immune system or all three ways simultaneously (Hakansson and Molin et al., 2011). *Faecalibacterium prausnitzii* from the family *Ruminococcaceae* has been reported to secrete anti-inflammatory metabolites that block the activation and secretion of NF-κB and IL-8 in Caco-2 cells (Sokol et al., 2008). Also, *F. prausnitzii* can induce an increase in the anti-inflammatory ratio of IL-10 and IL-12 (Alameddine et al., 2019). An endoscopic recurrence of Chron’s disease has been reported when there is a lower proportion of *F. prausnitzii* within the composition of the patient’s group (Sokol et al., 2008).

**FIGURE 3:**
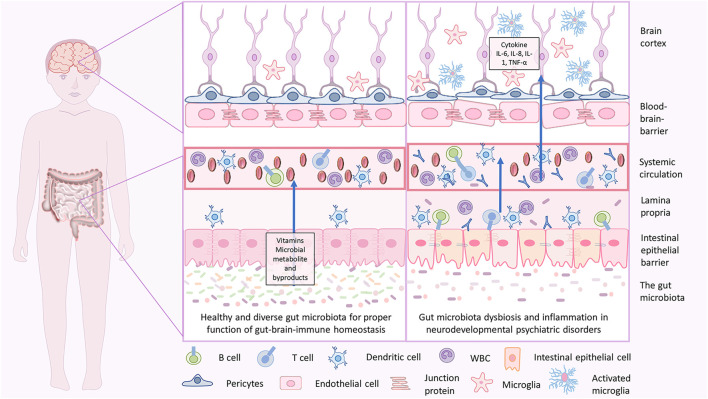
Gut-brain homeostasis and inflammatory mechanisms in neurodevelopmental psychiatric disorders due to gut brain dysbiosis. In infancy, the dysbiotic gut is characterized by less diverse microbiota with an abundance of pathogenic microbes, less beneficial microbes, and disrupted gut epithelial barrier with consequent GI tract-related disorders. When severe, the pathogenic microbes may cross through and enter the blood, which holds a massive immune response and releases inflammatory cytokines that lead to inflammation. Cytokine imbalance can induce microglial activation in the brain, which again causes neuroinflammation. This is associated with a disruption in brain development, and delay in neurodevelopmental subsequently may lead to NDD and NPDs.

**FIGURE 4:**
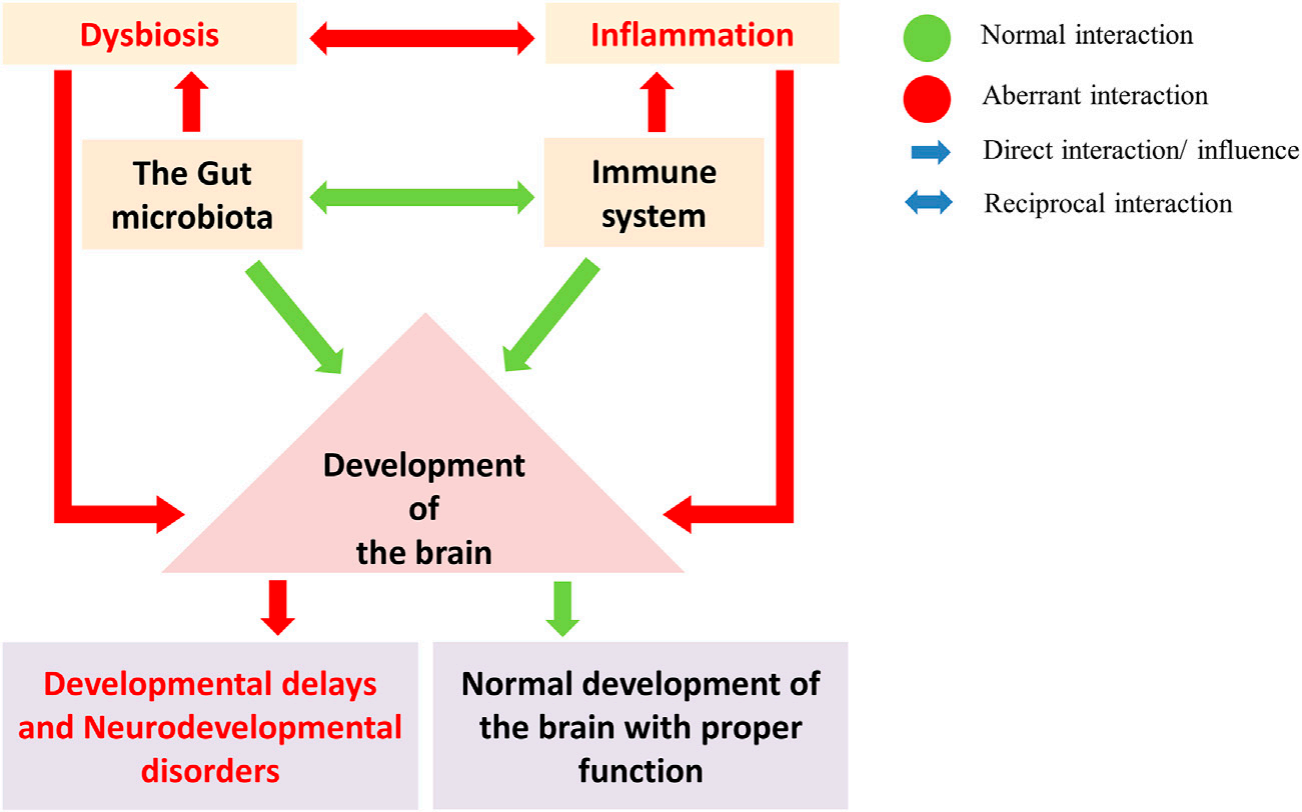
The gut microbes regulate the development and maturation of the immune system and play a crucial role in brain development (Green: normal interaction; Red: aberrant interaction). Gut dysbiosis and the immune system in brain development led to neurodevelopmental disorders. A gut dysbiosis alters the immune system homeostasis and leads to the activation of inflammatory response. Such aberration during brain development can disrupt the developmental pathways, leading to developmental delays and neurodevelopmental disorders.

Moreover, oral administration of *F. prausnitzii* can reduce the disease severity induced by 2, 4, 6-trinitrobenzene sulfonic acid in the colitis mouse model (Y. Zhou et al., 2021). *Lactobacillus* and *Bifidobacterium* are well-known probiotics and are the most studied taxa having anti-inflammatory properties and are the most crucial part of the gut microbiota (Cristofori et al., 2021). Microbes belonging to taxa *Lactobacillus* and *Bifidobacterium* have been reported to suppress an undesired activation of the immune response as in case of autoimmunity and allergy, while capable of inducing an increase in nonspecific IgA antibody secretion in the intestine (Sjögren et al., 2009; Kandasamy et al., 2015). Gut dysbiosis can be the consequence of an increase in inflammation-inducing microbes in the gut. Both dysbiosis and inflammation have a negative effect on brain development and can result in NDDs (Figures 3, 4).

### 4.2 The Gut Microbiota and Neurodevelopmental Disorders

Any alterations in the above neurodevelopmental process lead to a class of disorders affecting brain development and function that are collectively referred to as neurodevelopmental disorders, affecting 15% of the worldwide estimated population (Boyle et al., 2011). The gut microbiota has a significant role in neuronal development through a complex bidirectional communication, also known as the gut-brain axis. Alteration in the colonization of gut microbiota has a major impact on mammalian brain development that can influence the aberration in adult behavior by altering the expression pattern of several genetic events during the process (Heijtz et al., 2011). Also, the neurocrine disturbances (Clarke et al., 2013) and consequent behavioral phenotypes resulting upon disruption of the gut microbiota colonization during the developmental period cannot be reversed *via* reconstitution of microbiota at a later period (Sudo et al., 2004; Heijtz et al., 2011; Desbonnet et al., 2014). Inflammation-induced by dysregulation of the maternal immune system *via* maternal infection, maternal gut dysbiosis, or metabolic disorders during pregnancy has also been considered as modulating risk factors for abnormal brain development prenatally as well as neonatally, subsequently leading to a range of neurodevelopmental and neuropsychiatric disorders in the later period (Boulanger-Bertolus et al., 2018; Guma et al., 2021). Fetal exposure to maternal gut microbial products (propionic acid, LPS, or peptidoglycan), immune complexes, antibodies, and inflammation mediators can alter the normal neurodevelopmental process. Some of these specific factors are known to cross the placenta and the immature prenatal BBB and modulate several processes of brain development directly or by inducing neuroinflammation (Madore et al., 2016). Dysregulation of immune homeostasis and systemic inflammation share a direct connection with management complications of the gastrointestinal (GI) system (Chen et al., 2018), which eventually resulted in increased intestinal permeability (leaky gut) accompanied by various GI problems, and interestingly has a reciprocal correlation with gut microbial dysbiosis. Such GI complications have a reported association with various NDDs (Maguire and Maguire, 2019). After immediate birth among the first microbial colonizers, *Bifidobacteria* are the most important and dominant bacterial genera in the infant as well as adults’ gut commensals (Milani et al., 2017). Also, *Bifidobacteria* are considered among most potential probiotics and known to have anti-inflammatory, antimicrobial properties can utilize indigestible polysaccharides and can produce various B-group vitamins (O’Callaghan and van Sinderen, 2016). In addition, *Bifidobacteria* can reduce stress levels and help with depression by improving HPA axis stress response (Sudo et al., 2004), elevating the serotonergic precursor-tryptophan (Desbonnet et al., 2014), and having an anxiolytic effect (Bercik et al., 2011). Deficiency of intestinal *Bifidobacteria* has been linked with indigestion, vitamin B12 deficiency, dysregulated immune system, gut inflammation, depression, and anxiety-like behavior in individuals suffering from NDDs (Jolanta Wasilewska and Klukowski, 2015; Y.; Zhang et al., 2016). Moreover, in NDD patients, there is a significant decrease or complete absence of *Bifidobacteria*, while no such case was observed in any of the control subjects (Bojović et al., 2020). An association between the total SCFAs level and abundance level of *Faecalibacterium, Ruminococcus,* and *Bifidobacterium* was found (de Angelis et al., 2013), whereby *R. champanellensis* and *F. prausnitzii* are present in lower abundance in the patients, with several other strains from the genus *Bifidobacterium*. *Desulfotomaculum guttoideum*, *Romboutsia ilealis,* and *Intestinibacter bartlettii* are the microbial sp closely related to *Clostridium* clusters (Gerritsen et al., 2014) were commonly found in the NDD patients (Bojović et al., 2020). Moreover, microbes classified under genera *Bifidobacterium* and *Ruminococcus* cannot self-produce SCFA butyrate but can induce the production of SCFAs because of their ability to digest undigestible carbohydrates. They can induce butyrate production by providing substrates for butyrate-producing microbes in the colon area (Belenguer et al., 2006). SCFAs are important molecular intermediaries of the gut-brain axis, suggesting their involvement in developing autism-like symptoms (Rogers et al., 2016). Lower numbers of SCFA-producing bacteria, particularly the butyrate-producing bacteria in the affected individual’s gut, have been reported to exert a substantial impact on the etiopathology of NDDs (Bojović et al., 2020). One of the microbial species, namely *Dialister invisus*, *is* isolated from the oral cavity usually present in the gut microbiota of healthy humans (Downes et al., 2003), and a decrease in its abundance has been noticed in the NDD patient group (Bojović et al., 2020). This depicts that the variance in general commensal microbes in the NDD patients is significantly lower, with an abundance of potentially harmful bacteria than the healthy control group. Besides, people suffering from NDD commonly suffer from gut microbiota dysbiosis. However, there is a difference between the type of microbial richness and diversity within the gut microbiota and its association with different NDDs (Lacorte et al., 2019). Tables 1–3 provide a detailed summary of gut microbial abundance and altered cytokine levels in the case of NDDs.

**TABLE 1:** Gut microbes and Level of cytokines in autism spectrum disorder (ASD).

Neurodevelopmental disorders	Autism spectrum disorder (ASD)	
	Increased abundance	Decreased abundance
**Gut microbiota**	*Candida* sp. Iovene et al. (2017)	*Bacteroidetes* Finegold et al. (2010); Kang et al. (2013)
	*Clostridium histolyticum, Erysipelatoclostridium ramosum*, *Clostridium* sp. Finegold et al. (2002); Parracho et al. (2005); de Angelis et al. (2013)	*Ruminococcaceae, Streptococcaeceae, Peptostreptococcaceae, Erysipelotrichaceae, Faecalibacterium, Ruminococcus*, *Eubacteriaceae* de Angelis et al. (2013)
	*Clostridium tetani* Bolte (1998); Hsiao et al. (2013)	*Ruminococcaceae, Streptococcaceae, Peptostreptococcaceae, Erysipelotrichaceae* Bezawada et al. (2020); Zhang et al. (2018)
	*Clostridium bartlettii* Shattock and Whiteley, (2002)	*Alistipes, Dialister, Parabacteroides* Strati et al. (2017)
	*Desulfotomaculum guttoideum* Finegold et al. (2012a); Tomova et al. (2015)	*Streptococcus, Veillonella*, *Escherichia* Zhang et al. (2018)
	*Sutterella* B. L. Williams et al. (2012)	*Prevotella, Bifidobacterium* sp Finegold et al. (2010); Kang et al. (2013)
	*Fusobacterium, Barnesiella, Coprobacter, Olsenella, Allisonella*, *Actinomycetaceae*, *Holdemanella* Lacorte et al. (2019)	*Romboutsia timonensis* Yap et al. (2021)
	*Firmicutes* Finegold et al. (2010); Kang et al. (2013)	
	*Lactobacillaceae*, *Bifidobacteriaceae, Veillonellaceae, Enterobacteriaceae* Zhang et al. (2018); Bezawada et al. (2020)	
	Genus: *Bacteroides, Parabacteroides, Sutterella, Lachnospira, Bacillus, Biophila, Lactococcus, Lachnobacterium, Oscillospira, Megamonas*, *Bacteroides, Faecalibacterium, Bifidobacterium, Lactobacillum, Megasphera, Mitsuokella* Lacorte et al. (2019)	
	*Collinsella, Corynebacterium, Dorea* Strati et al. (2017)	
**Cytokines levels**	IL-8, TNF-α, IL1β Rodríguez et al. (2017)	Transforming growth factor β (TGF-β) IL-2 Ashwood et al. (2011)
	IL-8, IL-17 Suzuki et al. (2011)	IL-2 Eftekharian et al. (2018); Jácome et al. (2016); Molloy et al. (2006)
	IL-1β, IL-6, IL-12p40, TNF-α Ashwood et al. (2011)	
	IL-1β, IL-6, IL-17, IL-12p40, IL-12p70 Molloy et al. (2006); Jácome et al. (2016); Eftekharian et al. (2018)	
	IL-β, IL-6, IL-17, IL-18, IL-33, TNF-α Prata et al. (2017)	
	IL-1RA, IL-5, IL-8, IL-12p70, IL-13, IL-17, GRO-α Suzuki et al. (2011)	

**TABLE 2:** Gut microbes and level of cytokines in attention-deficit/hyperactivity disorder (ADHD).

Neurodevelopmental disorders	Attention-deficit/hyperactivity disorder (ADHD)	
	Increased abundance	Decreased abundance
**Gut microbiota**	Family: *Bacteroidaceae, Neisseriaceae* Lacorte et al. (2019)	Family: *Alcaligenaceae* Jiang et al. (2018)
	Family: *Peptostreptococcaceae*, *Moraxellaceae*, *Xanthomonadaceae*, *Peptococcaceae* Jiang et al. (2018)	*Bifidobacterium* Pärtty et al. (2015)
	Genus: *Faecalibacterium*, *Dialister*, *Lachnoclostridium*, *Sutterella* Prehn-Kristensen et al. (2018)	*Bacillus macerans* Clapp et al. (2017)
	Genus: *Bifidobacterium* Hiergeist et al. (2020)	*Bifidobacterium, Lactobacillus, Streptococcus* sp., *Enterococcus* sp Bundgaard-Nielsen et al. (2020)
	*Alistipe, Oscillibacter* Frémont et al. (2013); Naseribafrouei et al. (2014)	*Bacteroidales* Frémont et al. (2013); Naseribafrouei et al. (2014)
**Cytokines levels**	TNF-α Oades et al. (2010); Anand et al. (2017)	IL-4, IL-2, INF-γ Anand et al. (2017); Oades et al. (2010)
	IL-2, IL-6, IFN-γ, IL-10, IL-13, IL-16 Oades et al. (2010)	IL-1β Oades et al. (2010)
	IL-13, IL-16 Oades et al. (2010)	IL-2 Oades et al. (2010)
	IL-6, IFN-γ Oades et al. (2011)	TNF-α (Oades 2011)
	IL-6, IL-8, TNF-RI O’Shea et al. (2014)	

**TABLE 3:** Gut microbes and Level of cytokines in Schizophrenia (SCZ).

Neurodevelopmental disorders	Schizophrenia (SCZ)	
	Increased abundance	Decreased abundance
**Gut microbiota**	Phylum: *Proteobacteria* Genera: *Succinivibrio, Megasphaera, Collinsella, Clostridium, Klebsiella*, and *Methanobrevibacter* Shen et al. (2018)	*Coprococcus, Roseburia*, *Blautia* Shen et al. (2018)
		Phylum: *Proteobacteria,* Family: *Rumimococcaceae,* Genus: *Haemophilus*, *Sutterella*, *Clostridium* Nguyen et al. (2018)
	*Anaerococcus, Bacteroids* Nguyen et al. (2018)	
	*Veillonella atypica, Veillonella dispar, Bifidobacterium dentium, Dialister invisus, Lactobacillus oris*, *Streptococcus salivarius*, *Lactobacillus fermentum, Enterococcus faecium, Alkaliphilus oremlandii*, and *Cronobacter sakazakii/turicensis* Zhu et al., (2020a)	*Bifidobacterium, E. coli*, and *Lactobacillus* Cuomo et al. (2018)
		*Bifidobacterium longum* Katz-Barber et al. (2020)
		*Rhodocyclales*, *Enterococcaceae*, *Rikenellaceae*, *Alcaligenaceae*, *Rhodocyclaceae, Leuconostocaceae*, *Enterococcus* Xu et al. (2019)
	Taxa: *Lactobacillaceae, Lachnospiraceae, Veillonellaceae* Schwarz et al. (2018); Zheng et al. (2019)	Family: *Erysipelotrichaceae,* genus: *Allobaculum* Gubert et al. (2020)
	*Clostridiales, Lactobacillales, Bacteroidales* de Angelis et al. (2013); Shen et al. (2018)	
	*Akkermansia muciniphila, Bacteroides plebeius, Veillonella parvula, Clostridium symbiosum, Eubacterium siraeum, Cronobacter sakazakii/turicensis, S. vestibularis, Alkaliphilus oremlandii, Enterococcus faecium, Bifidobacterium longum*, *Bifidobacterium adolescentis* (Zhu et al., 2020a)	
	*Deltaproteobacteria*, *Actinobacteria*, *Sphingomonadales*, *Actinomycetales, Sphingomonadaceae*, *Megasphaera, Eggerthella* and, *Megasphaera elsdeniis*, *Clostridium perfringens, Akkermansia*, *muciniphila*, *Lactobacillus gasseri, and Bifidobacterium adolescentis* Xu et al. (2019)	
	*Acidaminococcus, Akkermansia, Alistipes, Citrobacter, Dialister, Veillonella* Zheng et al. (2019)	
**Cytokines levels**	C4A Stevens et al. (2007); Schafer et al. (2012); S.; Hong et al. (2016); Comer et al. (2020)	IL-1β, IL-10 Delaney et al. (2019); Park and Miller, (2020); Perkins et al. (2015)
	C-reactive protein (CRP) Prins et al. (2016); Ligthart et al. (2018)	IL-17 Zeni-Graiff et al. (2016)
	sIL-2R Rapaport et al. (1993); Gaughran et al. (2002); Martínez-Gras et al. (2012)	
	Interferon regulatory factor 3 (IRF3), interferon gamma (IFN-γ), interleukin 1 (IL)-1α, IL-1β, IL-6, IL-10, TNF-β, and TGF-β, CRP mRNA X. Li et al. (2015); Paul-Samojedny et al. (2011); Katila et al. (1999); Sasayama et al. (2011); Kalmady et al. (2014); Frydecka et al. (2015); Gao et al. (2014); Kroken et al. (2019)	
	IL-8 Ellman et al. (2010)	
	IL-1b Meisenzahl et al. (2001)	
	IL-6, IL-8, IL-10 Wu et al. (2019)	
	CRP, IL-6, IL-4, IL-12 Perkins et al. (2015); Delaney et al. (2019); Park and Miller (2020)	
	IL12/IL23p40 Föcking et al. (2016)	
	IFN-γ and IL-12, IL-1β and IL-10 Goldsmith et al. (2016); Lesh et al. (2018)	
	(IFN-γ), IL-1β, IL-1 receptor antagonist (IL-1RA), IL-6, IL-8, IL-10, IL-12, sIL-2R, transforming growth factor beta (TGF-β), and TNF-α Goldsmith et al. (2016)	
	IL-1 b, sIL-2R, IL-6, TNF-a Upthegrove et al. (2014)	
	IL-6, IL-1ß, CD14, and TNF-a Calderón-Garcidueñas et al. (2008); Calderón-Garcidueñas et al. (2015); Gruzieva et al. (2017)	

### 4.3 Role of Inflammation in Brain Development and Neurodevelopmental Disorders

Evidence suggests that dysregulated inflammation may be at the root of deficits that occur in numerous neurodevelopmental disorders, including schizophrenia and autism spectrum disorder (Ransohoff et al., 2015). Correlation between inflammation and NPDs in children has been gaining interest increasingly due to the harmful effects of inflammation on neuronal plasticity, neurogenesis, and overall neuronal development (Garay and McAllister 2010; Nona M.; Jiang, 2016). Inflammatory molecules affect brain development *via* interacting with MHC class-I molecule, glial cells, monoamine metabolism pathway, and HPA axis (Nona M. Jiang, 2016). In addition, immune cells, including B-cell, T cells, and macrophages, play a crucial role in the processes of brain development and are responsible for the onset of NPD (Shogo and Toshihide, 2018). Besides B-1a cell, one of the B-cell subtypes prevalently localizes to the neonatal brain in response to choroid plexus, reportedly involved in oligodendrocyte maturation in the developing brain (Shogo and Toshihide, 2018). However, in normal physiological conditions, B-1a cells preferentially localize in the intra-peritoneal cavity and participate in the neuro-immune defense system against any pathogenicity through the innate immune capability to secrete IgM antibodies, along with suppressing neuroinflammation, hence maintaining the neuroimmune homeostasis of the developing brain (Baumgarth, 2011; Kurnellas et al., 2015). Likewise, T-cells producing interferon-γ (IFN-γ) and IL-4 have been reported to regulate neuronal connectivity, development of cognitive, social behavior, and learning-related memory, respectively (Derecki et al., 2010; Filiano et al., 2016). The complete absence of T-cells in nude mice shows significant impaired neurogenesis and brain dysfunctionality (Ziv et al., 2006). Additionally, M2 macrophages subtypes are known to secrete neurotrophic factors such as IL-4, IGF-1, and upon induction BDNF, which promotes hippocampal neurogenesis, neuronal survival, and enhancement of spatial memory (Rőszer, 2015; Qi et al., 2017). IGF-1 and IL-4 are also produced by microglia and T cells, respectively (Derecki et al., 2010; Ueno et al., 2013). Also, perivascular macrophages support BBB development by providing pericytes progenitor cells (Daneman et al., 2010; Yamamoto et al., 2017).

Throughout the pregnancy, the maternal immune system and a fetal-placental immune response protect both mother and fetus in coordination with each other *via* systematic expression of various immune receptors that recognize diverse extra/intracellular pathogen and related products. And with pathogenic encounters, effectively produce a moderated inflammatory response, downstream signaling pathways related to pathogen clearance along with the secretion of antimicrobial peptides, and Indoleamine 2,3-dioxygenase (IDO) that are directly involved with pathogen clearance at the uterine-placental interface (Hoo et al., 2020). Thus, the immune system is likely to be present in an activated state rather than in the modulated state. And any case of immune alteration during this period can result in an increased susceptibility towards microbial and parasitic infections (Mor and Cardenas, 2010). This may trigger the fetal inflammatory response syndrome (FIRS) and results in an increased cytokine level of IL-1, IL-6, IL-8, and TNF-α, which has reported an increased risk of fetal developmental abnormalities (Gotsch et al., 2007). Moreover, human studies have also demonstrated a significant correlation between MIA, FIRs and the development of neurodevelopmental psychiatric disorders such as ASD, schizophrenia, neurosensorial deficits, ADHD, and psychosis in early as well as in the later period of adulthood (Knuesel et al., 2014; Brown and Conway, 2019; Choudhury and Lennox, 2021).

Further, research in the experimental mouse model has characterized high pro-inflammatory cytokine levels in the blood and amniotic fluid upon immune stimulation during pregnancy (Mor and Cardenas, 2010). It may likely contribute to the immunopathology of NDDs in their offspring. Certain inflammatory and immune-related conditions specific to particular NDD conditions, associated with inflammation induced by MIA, FIRS induction, nvolved in the development of NDD in offspring (Gotsch et al., 2007; Knuesel et al., 2014; Jung et al., 2020). Tables 1–3 provides a detailed summary of elevation and downfall in cytokine level during the inflammatory conditions in NDDs. The association of well-known neurodevelopmental-psychiatric disorders with inflammation and gut microbiome are discussed below.

### 4.4 Autism Spectrum Disorder

#### 4.4.1 The Gut Microbiome in ASD

Children with ASDs show a range of communication problems like lack of interest in social involvement, trouble expressing their feelings, and mostly avoiding physical contact, with trouble in speaking tendency (NIH, 2016). In addition, abnormality restrictive and repetitive behaviors, like aligning toys, flapping hands, swinging their body, or revolving in circles, have been the hallmarks of ASD (S. H. Kim and Lord, 2010). Around 80% of the children with ASD suffer from GI symptoms that include dyspepsia, abdominal pain, and constipation. The main reason for these severe GI problems could be the disruption of the gut microflora (Jolanta Wasilewska and Klukowski, 2015). In a recent study, restrictive food preference-based diet has also been linked with dysbiosis and decreased gut microbial diversity in children with ASD (Yap et al., 2021). Whilst the gut microbiome can regulate dietary preference, food choices as well as eating-related behavior, and a healthy gut microbiome is associated with healthy food choices (Alcock et al., 2014; de Wouters d’Oplinter et al., 2021; Koponen et al., 2021). Moreover, during pregnancy, dysbiosis of the maternal gut effectively alters gut-microbial diversity and immunity in the offspring, bringing an early onset of NDD (Nyangahu et al., 2018; Di Gesù et al., 2021). Additionally, altered immunity and inflammation also cause dysbiosis (Zeng et al., 2017).

Increasing evidence supports an association between gut microbiota dysbiosis and ASD (Xu et al., 2019). Through culture methods, aggressive forms of *Candida* species have been identified in the stool samples of 57% of children suffering from ASD compared to healthy controls (Iovene et al., 2017). Investigation of correlation between microbiome and ASD revealed that *Clostridium histolyticum,* a pathogenic anaerobic bacterium (*Clostridium* clusters I and II), was highly abundant in the fecal microbiota of ASD individuals. Several research groups have also detected an overgrowth of *Clostridium* sp. in the microbiota of children with autism (Finegold et al., 2002; Parracho et al., 2005; de Angelis et al., 2013). *Erysipelatoclostridium ramosum* was isolated from the fecal specimens of autistic children (Finegold et al., 2002). *Clostridium tetani* produce a neurotoxin p-cresol and other toxic metabolites (phenols and indole derivatives) that cause anxiety-like behavior (Bolte, 1998; Hsiao et al., 2013). *Clostridium bartlettii* is known to synthesize trans-3-indole acrylic acid (IAA), and by glycine conjugation, it can get converted into Indolyl-3-acryloyl glycine (IAG), which is a speculative ASD urinary diagnostic marker (Shattock and Whiteley, 2002; Bull et al., 2003). Overgrowth of these kinds of microbes has been associated with ASD pathology. Abnormality in sulfur metabolisms, such as the decline of serum sulfur in blood with a higher urinary excretion, decreased trans-sulfuration, and reduction in methylation capacity, in addition to chronic oxidative stress (Finegold et al., 2012b), have been detected in children with autism. This may be due to an increase in another sulfate-reducing organism, *D. guttoideum,* similar to *Desulfovibrio* (Finegold et al., 2012a; Tomova et al., 2015). In addition to *Chloristidium* cluster bacteria, *Sutterella*, associated with gut-immune homeostasis, and GI symptoms, has been found closely related with the intestinal epithelial layer among autistic children while absent in controls (B. L. Williams et al., 2012). Moreover, a particular relation between microbes *Actinomycetaceae, Allisonella, Barnesiella, Coprobacter, Fusobacterium, Olsenella* in ASD individuals and constipation have been observed. In contrast, there was an increase in *Holdemanella* in the case of ASD without associated constipation (Lacorte et al., 2019). The microbiome of ASD individuals represents a phylum level ratiometric increase in *Firmicutes*/*Bacteroidetes* due to a significant increase in *Firmicutes* and a reduction in the abundance of *Bacteroidetes* (Finegold et al., 2010; Kang et al., 2013)*.* A lower abundance of *Eubacteriaceae, Erysipelotrichaceae*, *Faecalibacterium*, *Peptostreptococcaceae*, *Ruminococcaceae*, and *Streptococcaeceae*, has been reported at a taxa level (de Angelis et al., 2013). At the family level, a considerably higher abundance of *Bifidobacteriaceae, Lactobacillaceae*, *Enterobacteriaceae,* and *Veillonellaceae* have been observed (Bezawada et al., 2020). At the genus level, there was a significant increase in *Bifidobacterium, Bacteroides, Bacillus, Biophila, Faecalibacterium, Lactobacillus, Lachnospira, Lactococcus, Lachnobacterium, Megamonas*, *Megasphera, Mitsuokella Oscillospira, Parabacteroides, Sutterella* (Lacorte et al., 2019), *Collinsella, Corynebacterium,* and *Dorea* (Strati et al., 2017)*.* A lower abundance of genera *Streptococcus, Veillonella*, *Escherichia* (Zhang et al., 2018), *Alistipes, Dialister, Parabacteroides* (Strati et al., 2017) were observed in autistic children. Additionally, Yap *et al.* reported a specific decline in *Romboutsia timonensis* abundance, especially in the gut microbiota of ASD children (Yap et al., 2021). Furthermore, SCFAs are crucial for maintaining BBB integrity and essential during the process of brain development (Silva et al., 2020), with proper maintenance of the functional intestinal epithelial barrier (Peng et al., 2009). SCFAs like butyrate, propionate and acetate solely or in combination with others can stimulate the tight junctions (TJ) formation (Ohata et al., 2005; Peng et al., 2007, 2009). Especially butyrate regulates the formation of BBB (Nankova et al., 2014). Further, butyrate and propionate exert a strong epigenetic effect over-regulation of the gene expression profile of neurotransmitter systems, neuronal cell adhesion molecules, inflammation, mitochondrial function, oxidative stress, and lipid metabolism. All these have been reportedly associated with the development of ASD (Nankova et al., 2014). Lower SCFAs levels may disrupt epithelial barrier function in the intestine, and BBB thus increases the permeability as reported in ASD patients (Fiorentino et al., 2016). Decreased SCFAs levels in the gut can be related to the absence of potential SCFA-producing bacteria in ASD patient microbiota (Bojović et al., 2020). Assisting all these reports, lower *Bifidobacterium* sp. levels were found in children with autism (Wang et al., 2011).

#### 4.4.2 Inflammation in ASD

Cytokines regulate immune response and are interestingly involved in neuronal development synaptic functions, differentiation, migration, proliferation, and behavioral impairments (Deverman and Patterson, 2009; Mousa and Bakhiet, 2013). Neuropoietic cytokines such as IL-6, IL-1β, and TNF-α have been reported to exert direct influence on the cortical neuron, dendrite development, neural activity, long-term potentiation, neurite outgrowth, regulation of synaptic plasticity in the hippocampus, overall neurodevelopment as well as behavior (Deverman and Patterson, 2009; Mousa and Bakhiet, 2013). Dysregulation in the cytokine levels creates disturbances in immune homeostasis, which potentially contribute to communication and behavioral impairment at the neuronal level in early childhood and, upon becoming severe, can give rise to NDDs like ASD (Goines and Ashwood, 2013; H. K.; Hughes et al., 2018). In children with ASD increased number of monocytes and plasma concentrations of IL-8, TNF-α, IL1β are observed, which indicate an abnormal inflammation condition (Rodríguez et al., 2017). Increased plasma concentration of IL-8 has been reported as a result of increased IL-17 released by activated Th_17_ cells in response to epithelial and endothelial infections (Suzuki et al., 2011). Immune dysfunction in ASD patients like abnormal T helper cell profile (Ashwood and Wakefield, 2006), increased concentration of complement factors (Corbett et al., 2007), pro-inflammatory interleukins (IL-1β, IL-6, IL-12p40) (Ashwood et al., 2011), TNF-α, and a decreased level of Transforming growth factor β (TGF-β) have been reported to cause acute inflammation analogous to ASD condition. Abnormal cytokine levels have been associated with poor health and communication, impaired social interaction, poor cognition/memory, and behavioral and neuronal dysfunction in ASD (Ashwood et al., 2011; Tye et al., 2019). Further, more studies conducted in children with ASD have revealed an elevated plasma level of IL-1β, IL-6, IL-17, IL-12p40, and IL-12p70, including both Th1 and Th2 cytokines, with down-regulation of IL-2 (Molloy et al., 2006; Jácome et al., 2016; Eftekharian et al., 2018). There also has been a report suggesting a sex-specific correlation of cytokine expression profile. According to which the mRNA expression level of TNF-α has been linked with expression of TGF-β, INF-γ, IL-17, and IL-6 in the case of males, whereas it's not the case with the females (Eftekharian et al., 2018). Besides, reports also show a difference in cytokine expression profile as per the disease severity in children with ASD. At a mildly severe condition, there is an elevation in plasma level of IL-12p40. In contrast, patients with moderate disease severity have higher plasma levels of TNF-α, which explains the correlation of disease severity with increased plasma concentration of TNF-α (Jácome et al., 2016; Xie et al., 2017).

In addition to all these inflammatory factors that contribute to ASD pathology, dysregulation in the maternal immune system during pregnancy has also been implicated in the development of ASD (Bilbo et al., 2018). Fetal brain reactive antibody, autoimmunity, or IgG antibody from mother can cross the placental barrier, thus entering into the fetal compartment where they can interfere with the developmental process by recognizing self-proteins. Also, in the fetus brain, the BBB is not fully formed or functional (Croen et al., 2008; Braunschweig and van de Water, 2012; Fiorentino et al., 2016). Therefore, the probability of immune-complexes and inflammatory mediators in action can reach the brain stem by crossing the BBB, contributing to microglial activation and neuronal inflammation eventually (Morgan et al., 2010; Fiorentino et al., 2016). Neuroinflammation within the CNS is also associated with severe disease conditions in ASD (Dipasquale et al., 2017; Eissa et al., 2020). Animal and human studies have suggested an elevated IL-6 expression in the autistic brain, causing anatomical aberration. Such overproduction of IL-6 can alter synapse formation and neurotransmission along with distorted patterns and distribution of dendritic spines (Wei et al., 2011, 2012, 2013). Moreover, the ASD animal model studies showed systemic inflammation with up-regulated IL-β, IL-6, IL-18, IL-33, IL-17, and TNF-α along with microglia activation and neuroinflammation principally contributing to the pathological mechanisms of ASD (Prata et al., 2017).

### 4.5 Attention-Deficit/Hyperactivity Disorder

#### 4.5.1 The Gut Microbiome in ADHD

Attention-deficit/hyperactivity disorder is commonly characterized by persistent inattention and hyperactivity-impulsivity onset during childhood. Genetic factors (Faraone et al., 2005), streptococcal infection (Leslie et al., 2008), and environmental factors (Mulligan et al., 2013; Guney et al., 2015) have a close association with ADHD. However, dopamine (DA) deficiency and an altered level of norepinephrine (NE), serotonin, GABA has also been proposed for ADHD (Blum et al., 2008; Wilens and Spencer, 2010), including a decline in cortisol level, indicating disruption of the HPA-axis as well (Fortier et al., 2013). The microbiota has an important role in the development of the HPA-axis, and individuals with ADHD experience an imbalance in their microbiota and a reduced microbial diversity and composition, which resulted in lower levels of cortisol to cause improper management of the HPA-axis stress response (Huo et al., 2017; Checa-Ros et al., 2021). Children with ADHD unusually have a higher load of the family *Bacteroidaceae, Neisseriaceae* differing from a higher level of *Prevotellaceae, Catabacteriaceae,* and *Porphiromonadaceae* in the control group. An increased microbial load of family *Neisseriaceae* and *Bacteroidaceae* has been reported causing a significant decline in the gut microbial diversity in ADHD (Lacorte et al., 2019). At a family level, the ADHD-specific fecal microbiome represents a substantial rise in the richness of *Moraxellaceae*, *Peptostreptococcaceae*, *Peptococcaceae*, *Xanthomonadaceae*, and a firm decline in *Alcaligenaceae* (Jiang et al., 2018). While, at a genus level, *Dialister*, *Faecalibacterium*, *Lachnoclostridium*, and *Sutterella* were the major representatives that brought the differences among children with and without ADHD, respectively (Prehn-Kristensen et al., 2018). Besides, a certain group of gut microbiota can synthesize neuroactive monoamine molecules (dopamine, noradrenaline, serotonin, GABA) and its precursors (phenylalanine, tyrosine, tryptophan) that are involved in ADHD pathomechanism. These microbes also induce intestinal epithelial cells to synthesize neuroactive compounds and indirectly control the neurotransmission system to have a crucial impact on brain functioning and behavior (Strandwitz, 2018; Y.; Chen et al., 2021; Huang and Wu, 2021). In a study including 28 participants, functional magnetic resonance imaging (fMRI) revealed a nominal increase in the genus *Bifidobacterium* in ADHD (Hiergeist et al., 2020). This increase has a significant association with enhanced bacterial gene functionality that encodes the enzyme cyclohexadienyl dehydratase (Aarts et al., 2017), an enzyme involved in the phenylalanine (a dopamine precursor) synthesis pathway (Lou, 1994). Phenylalanine can cross the BBB and regulate dopamine synthesis positively or negatively by inhibiting tyrosine hydroxylase during dopamine synthesis (Lou, 1994). Dopamine deficiency can cause decreased neural responses to reward anticipation, which is one of the hallmarks of ADHD (Aarts et al., 2017). On the other hand, diminished *Bifidobacterium* population during early childhood has been correlated with a greater risk of developing ADHD (Pärtty et al., 2015). *Bacillus macerans* can synthesize dopamine, as it possesses an enzyme called cyclomaltodextrin glucosyltransferase or CTGase, a key enzyme to produce dopamine (Clarke et al., 2013). Deficiency and decrease in abundance of such microbes in gut microbiota may contribute to stress, depression, and anxiety-like behavior that has been witnessed in ADHD (Clapp et al., 2017). In individuals with ADHD, altered serotonin levels have been linked with abnormal cognitive function and various other processes during neuronal development (Blum et al., 2008). Studies have demonstrated that gut microbes regulate the serotonin (5-HT) levels in the colon and blood, while the GI tract contains the majority of total serotonin present in the body either as 5-hydroxytryptamine or 5-HT. Microbes in the colon produce certain metabolites that stimulate the chromaffin cells to increase Tph levels and 5-HT biosynthesis (Huang and Wu, 2021). The serotonergic system resolves brains involvement in stress, anxiety, and depression (Graeff et al., 1996). Specific gut bacteria can produce important inhibitory neurotransmitters gamma-aminobutyric acid (GABA). Moreover, *Bifidobacterium* and *Lactobacillus* can produce GABA, while *Streptococcus* sp. and *Enterococcus* sp. which produce serotonin, are less abundant in the fecal microbiota of ASD and ADHD patients (Bundgaard-Nielsen et al., 2020). Another study showed an increase in *Alistipes* and *Oscillibacter* and a significant decrease in *Bacteroidales* in depressed individuals (Frémont et al., 2013; Naseribafrouei et al., 2014). *Bacteroidetes* has been associated with obesity previously, which is again associated with depression and mild inflammatory conditions (Chen et al., 2018). In addition, the genus *Oscillibacter* produces a metabolic end-product valeric acid, which is structurally similar to gamma-Amino Butyric acid (GABA). As a structural homolog of GABA, it can also bind to GABA receptors (Katano et al., 2012), facilitating increased inhibitory neurotransmission. This signifies the correlation of intra-microbiome coordination with depression and anxiety-like behavior in NDDs such as ADHD.

#### 4.5.2 Inflammation in ADHD

ADHD is reported to be immune-associated with an acute inflammatory response that emerges during childhood. Immune-associated genetic factors and heritability have been the most common cause of ADHD, estimated at around 70–78% (Franke et al., 2012). Most potentially genetic polymorphism of cytokine genes IL-6 and tumor necrosis factor-alpha (TNF-α) has been found in ADHD patients (Drtilkova et al., 2008). Moreover, cytokines play a significant role in tryptophan metabolism and dopaminergic pathways in the brain (Miller et al., 2013). Therefore, in the case of ADHD, an alteration in proportional expression of pro-inflammatory and anti-inflammatory cytokine can alter the required level of monoamine neurotransmitter (Dunn et al., 2019). Administration of IL-6, IL-2, IL-1β caused altered neurotransmission in the rodent model due to increased norepinephrine and decreased dopamine level, resembling the ADHD condition (Zalcman et al., 1994; Anisman et al., 1996). Also, in ADHD patients, there is an increased expression of innate pro-inflammatory cytokines like tumor necrosis factor-α (TNF-α) and a reduction in expression of anti-inflammatory cytokines like IL-4, IL-2, and INF-γ. These pro and anti-inflammatory cytokine alterations can induce microglial activation and neuroinflammation (Oades et al., 2010; Anand et al., 2017). The activated microglia release more pro-inflammatory cytokines and other associated factors, contributing to chronic neuroinflammation (Harry and Kraft, 2008). During the prenatal period, the peripheral and neuroinflammation alone or in combination may interfere with the maturation of the prefrontal cortex and neurotransmitter system, such as the dopaminergic system, which increases the risk of developing ADHD in offspring. Dopamine deficiency in CNS is one of the well-reported pathogenesis hallmarks of ADHD (Blum et al., 2008; Aarts et al., 2017). Moreover, allergic diseases that are considered high inflammatory disorders are also reported to be associated with ADHD (Wang et al., 2011; Miyazaki et al., 2017).

### 4.6 Schizophrenia

#### 4.6.1 Schizophrenia and Gut Microbes

SCZ is a chronic, heterogeneous neurodevelopmental psychiatric disorder with multifactorial involvement of genetic and epigenetic disturbances, gut microbiome alteration, immune system dyshomeostasis, and environmental influence correlated with hallucinations, delusional thoughts, perturbing mental awareness, and social interaction (Van Os and Kapur 2009). The disorder commonly involves continuous or intermittent occurrences of paranoia (Owen et al., 2016), with positive, negative, and cognitive psychotic symptoms. The positive symptoms include hallucinations, unsystematic speech patterns, and cataleptic behavior based on the excessiveness of normal function. In contrast, the negative symptoms involve a reduction in normal physiological processes, such as lack of emotions or interest, poor speech, aimlessness, and cognitive impairments, which comprise poor retention of verbal information and confusion (Munawar et al., 2021). SCZ is thought to begin *in utero* and is associated with prenatal famine, poor pregnancy, fetal growth conditions, emergency C-section delivery, and low birth weight (King et al., 2010). The functionalities of bidirectional gut-brain communication are related to emotional and cognitive centers such as the central nervous system, peripheral nervous system, enteric nervous system, and enteroendocrine system. Dysregulation might hold implications over the occurrence and etiology of SCZ (Grenham et al., 2011; Rogers et al., 2016).

As in ASD, Schizophrenic individuals suffer high rates of GI problems like gastroenteritis, colitis, irritable bowel syndrome (IBS) (Garakani et al., 2003; Shah et al., 2012; Yeh et al., 2021) and are mostly at high risk for emergence of hyperglycemia/diabetes, obesity, hypertension, and cardiovascular disease. Such metabolic disorders are strongly associated with microbiota dysbiosis, including neuropsychiatric disorders such as SCZ (de Hert et al., 2009; Ventriglio et al., 2015). Furthermore, the comorbidities of metabolic disorders in SCZ and enrichment of the specific gut microbes may disrupt brain white matter (Spangaro et al., 2018), causing the manifestation of negative symptoms and cognitive functional anomalies (Socała et al., 2021). Alteration in the gut microbiota is associated with a decreased species diversity within the microbiota in SCZ (Patrono et al., 2021).

There is an increase in the abundance of oropharyngeal microbial species like *Bifidobacterium dentium, Lactobacillus oris*, *Veillonella atypica, Dialister invisus, Veillonella dispar,* and *Streptococcus salivarius* in schizophrenic individuals than in healthy controls (Zhu et al., 2020; Castro-Nallar et al., 2015). Whereby the increased abundance of genera *Streptococcus* and *Veillonella* have positive cross-correlation, which also indicates a close association between the intestinal and the buccal microbiota in SCZ (Zhu et al., 2020). The increased translocation of oral microbe can be linked with disrupted the mucosal barrier, giving rise to leaky gut, pathological intestinal conditions, and decreased immune surveillance towards foreign microbes in SCZ patients (Zhu et al., 2020). Schizophrenic patients harbor a unique combination of facultative anaerobes, including *Lactobacillus fermentum, Alkaliphilus oremlandii*, *Enterococcus faecium,* and *Cronobacter sakazakii/turicensis,* that are usually not present in a healthy gut (Zhu et al., 2020).

An elevated phylum level of *Proteobacteria* characterizes the schizophrenic microbiome. Particularly, with a substantial rise in genera *Methanobrevibacter, Clostridium, Collinsella, Succinivibrio, Klebsiella*, *Megasphaera,* with a decrease in *Coprococcus, Blautia*, and *Roseburia*, which also has been associated with disturbed metabolic pathways like fatty acid, vitamin B6 metabolism in SCZ (Shen et al., 2018). Contrarily a decline in phylum Proteobacteria, family *Rumimococcaceae*, genus *Haemophilus*, *Sutterella*, and *Clostridium* have been reportedly associated with disease progression. In contrast, increased *Anaerococcus* and *Bacteroids* has also been reported in such schizophrenic conditions. The relative decrease in *Rumimococcaceae* is directly related to negative symptoms, and increased *Bacteroids* have been reported in association with the increase in the symptoms of depression in SCZ patients (Nguyen et al., 2018). Moreover, in the early stages of the disease, an increase in *Lactobacillaceae* (Schwarz et al., 2018), and at a later stage, bacterial taxa *Lachnospiraceae* and *Veillonellaceae* have been linked with the disease severity in SCZ (Zheng et al., 2019). Higher *Clostridiales, Lactobacillales, Bacteroidales* suggest an overlap between altered microbiome with those identified in SCZ and ASD (de Angelis et al., 2013; Shen et al., 2018).

Moreover, the increase in diversity of the microbes in the blood can be related to the indefinite overall increase of microbial load in SCZ (Olde Loohuis et al., 2018). The blood microbiota is thought to emerge from the gut and the buccal cavity (Potgieter et al., 2015; Spadoni et al., 2015). Schizophrenic patients suffering from GI inflammation and IBS have been reported to have elevated levels of anti-Saccharomyces cerevisiae antibodies (ASCA), used for diagnosis of Crohn’s disease (Desplat-Jégo et al., 2007; Rodrigues-Amorim et al., 2018). Zhu and co-workers reported 11 bacterial species (Table. 3) that were significantly enriched and have been particularly associated with symptom severity and poor cognitive performance in SCZ (Zhu et al., 2020). Again, the frequency of *Clostridium difficile* infection has been highly related to the schizophrenic patient population (Argou-Cardozo and Zeidán-Chuliá, 2018). Zheng et al. reported an increased abundance of *Acidaminococcus, Akkermansia, Alistipes, Citrobacter, Dialister, Veillonella* in SCZ (Zheng et al., 2019). In addition, a metagenomic study in SCZ patients revealed a strong association of 12 increased taxonomic abundant groups including *Deltaproteobacteria*, *Actinobacteria*, *Sphingomonadales*, *Actinomycetales, Sphingomonadaceae*, *Megasphaera, Eggerthella* and, *Megasphaera elsdeniis*, *Clostridium perfringens, Akkermansia*, *muciniphila*, *Lactobacillus gasseri, and Bifidobacterium adolescentis,* along with 7 decreased taxonomic groups including *Rhodocyclales*, *Enterococcaceae*, *Rikenellaceae*, *Alcaligenaceae*, *Rhodocyclaceae, Leuconostocaceae*, and *Enterococcus* in comparison to age and sex-matched healthy controls (Xu et al., 2020).

A decrease in diversity of family *Erysipelotrichaceae* and genus *Allobaculum*, along with a dysbiosis, has been reported in the metabotropic glutamate receptor 5 (mGlu5) knockout mice model of SCZ (Gubert et al., 2020). Further, a significantly low level of *Bifidobacterium, Lactobacillus, E. coli*, and a higher level of *Clostridium coccoides* were reported in fecal samples of SCZ individuals compared to healthy subjects (Cuomo et al., 2018). However, treatment with either olanzapine or risperidone was reported to alter the level of *Akkermansia, Sutterella*, and *Lachnospiraceae* with a considerable increase in *Bifidobacterium*, *E. coli* and a decrease in fecal *Clostridium coccoides* and *Lactobacillus* compared to untreated (Flowers et al., 2017; Yuan et al., 2018). Studies have shown that prenatal and inherent microbial infections in neonates can increase the risk of developing SCZ (Babulas et al., 2006; Brown and Derkits, 2010). Infection by *Helicobacter pylori* is associated with nutrient malabsorption during childhood and induces disruption in biochemical homeostasis, which may play an essential role in the pathogenesis of SCZ (Yilmaz et al., 2008; Vitale et al., 2011). *Toxoplasma gondii* infection during pregnancy is positively associated with SCZ in offspring (Brown et al., 2005). Moreover, *Streptococcus vestibularis* has been considered a microbial biomarker of SCZ that can induce deficits in social behaviors, alteration in neurotransmitter levels (Zhu et al., 2020), hyperactivity, cognitive impairments associated with SCZ (Maes et al., 2019).

SCZ is associated with the aberration of various physiological pathways involving dopamine, glutamate, and γ-aminobutyric acid (GABA) signaling (Rowland et al., 2016; Hoftman et al., 2018; Kesby et al., 2018), synthesis of SCFAs like butyrate, acetate, propionate, and isovaleric acid, tryptophan metabolism, and synthesis of several neurotransmitters (glutamate, GABA, and nitric oxide) (Zheng et al., 2019). Alteration of which are associated with symptoms, such as depression, anxiety, and pathophysiology of SCZ (Maas et al., 1993; Möhler, 2012; Erhardt et al., 2017), which again can be related to the alteration of gut microbial diversity in SCZ. Experimental, fecal transfer from SCZ patients into the GF mice gut induces SCZ-related behaviors resembling glutamatergic hypo-functionality related SCZ mice model behavior in receiver mice with observably distorted glutamate, glutamine, and GABA ratio in the hippocampus region of the brain (Zheng et al., 2019).

Aberration in glutamatergic signaling system-related genes is closely associated development of SCZ. Meanwhile, Glutamate synthase (GOGAT), validated as SCZ gut marker, an increase in expression and activity in SCZ patients is co-related with a characteristic decrease in gut microbial diversity and an altered I_g_A related mucosal immunity as compared to healthy controls (Gubert et al., 2020; Xu et al., 2020).

Tryptophan metabolism is one of the essential pathways for the maintenance and homeostasis of gut microbiota and disturbances have been reported to contribute to the pathophysiology of SCZ (Erhardt et al., 2017; Plitman et al., 2017). Tryptophan is metabolized chiefly through the kynurenine pathway, and schizophrenic individuals are reported to have high serum kynurenine metabolites and low serum tryptophan levels (Zhu et al., 2020; Muneer, 2020). A study involving fecal microbiota transplantation from drug-free SCZ patients into SPF mice reported an alteration of the kynurenine metabolic pathway and schizophrenic behavior in receiver mice. This alteration in tryptophan and kynurenine pathway metabolite level is directly associated with the gut-microbiota alteration and enrichment of schizophrenic specific microbes (Zhu et al., 2020). The translocation of microbes along with microbial components like LPS into the bloodstream due to gut dysbiosis as well as increased gut epithelial barrier integrity may be inducing systemic inflammation, that consequently leads to neuroinflammation, neurological impairment, and apoptosis, leading to immune-mediated development of SCZ (Fasano, 2012; Alhasson et al., 2017; Yuan et al., 2019). In addition, SCFAs have a direct role in anxiety and behavioral changes such as antisocial, repetitive, ritualistic behaviors associated with neuropsychiatric disorders like obsessive-compulsive disorder hyperactivity in SCZ (MacFabe, 2012). Furthermore, the reported elevation in microbial translocation marker sCD14 has been correlated with a reduction in butyrate-producing species *Roseburia* and *Coprococcus* with increased inflammation and CNS infections (Machiels et al., 2014). SCFAs also indirectly regulate DNA repair mechanisms by reducing the excessive activity of histone deacetylases (HDACs) in schizophrenic conditions, and reduced levels can negatively affect the diseased condition (Oleskin and Shenderov, 2016), suggesting an additional indirect role of SCFA in SCZ.

#### 4.6.2 Inflammation and Schizophrenia

SCZ affects approximately 1% of the worldwide population (Kahn et al., 2015). The complex interaction between genetic and environmental risk factors manipulates the immune system to cause inflammation, disruption of neuroimmune homeostasis, chronic neuroinflammation, and neurodevelopmental disturbances in SCZ (Comer et al., 2020). The risk factors include genetic anomaly, MIA, maternal infection, gut dysbiosis, early-life stress, and exposure to pollution, resulting in the dysfunctional BBB and microglial function that leads to neuroinflammation that gives rise to the onset and progression of SCZ (Comer et al., 2020). The characteristic brain damage in SCZ starts in the early periods of life manifests later in the lifetime (Altamura et al., 2013). The disease symptoms usually appear in late adolescence and early adulthood (Na et al., 2014).

Genome-wide association studies have revealed genetic polymorphism of immune-related genes in SCZ etiopathophysiology. For example, genetic polymorphisms of C4 complement factor, major histocompatibility complex (MHC) locus on chromosome 6 (Shi et al., 2009; Sekar et al., 2016), interleukin-1β, IL-6, the soluble IL-6 receptor (sIL6R), and IL-10 are associated with increased risk of SCZ (Xu and He, 2010; Gao et al., 2014; Shibuya et al., 2014; Hudson and Miller, 2018). In contrast, polymorphism of IL-2, IL-4, tumor necrosis factor-α (TNF-α), or transforming growth factor- β1 (TGF-β1), are not associated with increased risk of SCZ (Qin et al., 2013; Hudson and Miller, 2018). In addition, CD19 and CD20 genes associated with B-cell the onset of SCZ (Ripke et al., 2014). Genetic aberration in oligodendrocyte and myelination-associated genes also have been detected in schizophrenic patients (Katsel et al., 2005). Moreover, a significant rise of CD5^+^ B-cell is reported blood of schizophrenic individuals (Printz et al., 1999). This suggests a crucial contribution of B-cell functionalities in immunity, oligodendrogenesis, and myelination during brain development. A B-cell dysfunctionality was reported in pathophysiology and increased vulnerability in SCZ (Steiner et al., 2010; Birnbaum and Weinberger, 2017).

However, a higher blood level of acute-phase reactant CRP is negatively correlated with a decreased risk of SCZ (Prins et al., 2016; Ligthart et al., 2018), and higher soluble IL-2 receptors (sIL-2R) levels indicate an increased risk of schizophrenic events (Gaughran et al., 1998). Increased expression of complement protein C4A involved in microglia-mediated synaptic pruning has been reported to reduce connectivity/sociability, which contributes directly to pathophysiology in SCZ (Stevens et al., 2007; Schafer et al., 2012; Comer et al., 2020). In addition, variation in structural alleles of C4 poses an increased risk for autoimmunity and sex-specific vulnerability in SCZ in males (Kamitaki et al., 2020). A blood spot study on 892 newborns revealed increased C4A complement protein levels; those later developed SCZ (Cooper et al., 2017). CUB and sushi multiple domains 1 (CSMD1), expressed at the time of early perinatal development, which regulates C4 protein expression and a genetic disruption or pathway dysregulation may lead to deficits in average cognitive aptitude and management ability in SCZ (Kraus et al., 2006; Athanasiu et al., 2017).

Any deviation in expression of cytokines involving elevation in interferon regulatory factor 3 (IRF3) (crucial transcription factor during viral infection) (X. Li et al., 2015), IFN-γ (regulates viral multiplication) (Paul-Samojedny et al., 2011), pro-inflammatory IL-1α (Katila et al., 1999), IL-1β (Sasayama et al., 2011), IL-6 (Kalmady et al., 2014; Frydecka et al., 2015) and anti-inflammatory IL-10 (Gao et al., 2014), along with elevated mRNA levels of CRP, IL-6, IL-1β, TNF-β, and TGF-β, have been reported in SCZ individuals (Kroken et al., 2019). Immune receptors like MHC receptors that are involved in immune surveillance and Toll-like receptors (TLRs) that engaged in cognition of microbe-derived molecular signals by innate immune cells and microglia, early brain development (Mallard, 2012; C. Y.; Chen et al., 2019), synaptic plasticity and neurogenesis (Barak et al., 2014) have been found altered (Kang et al., 2013; García-Bueno et al., 2016; MacDowell et al., 2017) in either the blood or post-mortem brain tissue of SCZ individuals. A disruption in gene disrupted-in-schizophrenia- 1 (DISC1) was discovered in a Scottish family affected with SCZ (St Clair et al., 1990) and later worldwide (Chubb et al., 2008). Such genotypes disrupt the immune system network, thereby inducing inflammation (Brandon et al., 2009), suggesting a dysfunctional immune-related gene and inflammation co-ordinately contributes to the pathophysiology of SCZ (Trépanier et al., 2016; van Kesteren et al., 2017). MIA during pregnancy has reportedly caused an increase in mesencephalic dopaminergic neurons in the foetal brain, which is associated with excessive dopaminergic signaling in the midbrain area in SCZ (Winter et al., 2009). Besides, *in utero* MIA precondition offspring exhibit, a triggered HPA-axis stress response when exposed to cannabinoids in adolescence and exhibit SCZ associated behavior in a sex-specific manner with a remarkable decline in *Bifidobacterium longum* gut abundance (Katz-Barber et al., 2020). Exposure to viral or bacterial pathogens *in-utero* also has been reported to enhance the risk of developing SCZ. Maternal infection with *Toxoplasma gondii,* influenza, rubella, and Borna disease virus intensely increased the risk factor for SCZ (Brown et al., 2000; Brown et al., 2005). Moreover, autoimmune disorders (Benros et al., 2011), prenatal infections, and childhood exposure to different viruses (Brown et al., 2000; Pearce, 2001; Brown et al., 2004; Buka et al., 2008), *Toxoplasma gondii* infection (Brown et al., 2005) respiratory infections (Sørensen et al., 2009), genital or reproductive tract infections, (Babulas et al., 2006), and other infections (Gattaz et al., 2004; Dalman et al., 2008), have been reported to induce, behavioral and cognitive dysfunction and overall increased risk of development of SCZ in the offspring (Fineberg and Ellman, 2013; Na et al., 2014).

Unmedicated SCZ patients have a specific cytokine expression pattern with an abrupted type 1 IFN-γ, IL-2, sIL-2R, and a complimentary increase in type 2 cytokine pattern IL-6 and IL-10, indicating an imbalanced type 1 and type 2 immune responses in SCZ (Müller and Schwarz, 2006). An increased IL-8 level during the prenatal period is also associated with decreased brain cortex volume and increased ventricular volume in the schizophrenic progeny (Ellman et al., 2010). In addition, genetic risk for elevated expression of the immune marker IL-1β has been linked to brain volume loss (Meisenzahl et al., 2001). A recent study in SCZ patients has revealed increased serum cytokine levels of IL-6, IL-8, and IL-10, substantially associated with the cortical volume in the orbital frontal cortex, cingulate cortex, and frontotemporal gyrus (Wu et al., 2019). Increased level of IL-6 is associated with resurfacing SCZ associated factors preconditioned before birth (D. Zhou et al., 1993). Also, IL-6 has a strong influence on the decreased survivability of serotonergic neurons in the fetal brain (Jarskog et al., 1997), and IL-1β has been reported to induce the differentiation of mesencephalic progenitor cells into dopaminergic neurons in the animal model (Kabiersch et al., 1998; Potter et al., 1999). This is relatable to the potential impact of cytokines on the establishment of neurotransmitter systems in schizophrenic conditions. Further, higher blood levels of IL-6 in early life have been associated with an increased risk of experiencing psychotic episodes with manifestations of fulminant psychotic disorder at age 18 (Khandaker et al., 2014). IL-6 and IL-17 may bring on the poly (I: C), an immune stimulant in the MIA model, which affects the fetal brain (Choi et al., 2016; S. E. P.; Smith et al., 2007). Studies show associations of higher CRP levels with elevated serum IL-6 with a significantly altered cognition in chronic and first-episode SCZ, along with poor verbal and learning memory, poor management skills, and disrupted psychomotor speed (Miller et al., 2013; Misiak et al., 2018). Also, CRP levels have a significant positive correlation with BMI. In contrast, a negative correlation with HDL levels in psychosis periods (Carrizo et al., 2008) and higher CRP levels are associated with increased waist circumference in males compared to sex as well as age-matched patients (Fawzi et al., 2011). In addition, higher IL-6 and hs-CRP levels were notably positively correlated with BMI only in females (C. W. Kelly et al., 2019). Psychotic patients with an increased CRP level are most likely to have metabolic syndrome (Vuksan-Ćusa et al., 2010; Boyer et al., 2013). A meta-analysis revealed that increased inflammatory marker CRP, IL-8, and IL-10 in maternal blood during pregnancy, associated with higher risks, along with an early sign and symptoms in SCZ, suggesting prenatal inflammation alter the brain development process in the fetus, with increased susceptibility to SCZ (Zhang et al., 2018). CRP has essentially been correlated with negative symptoms and psychopathology, whereas IL-18 has been positively associated with total and general psychopathology in SCZ (B. Miller and Essali, 2019). In clinically high-risk patients, there has been a reported increase in CRP, IL-6, IL-4, IL-12, and a decrease in IL-1β, IL-10 (anti-inflammatory cytokine) (Perkins et al., 2015; Delaney et al., 2019; Park and Miller, 2020). A higher concentration of IL-12/IL-23p40 was detected in particulars who started to experience psychosis (Föcking et al., 2016).

In Patients with the first episode of psychosis (FEP), considerably higher levels of IFN-γ and IL-12 were inversely correlated with whole-brain grey matter (Lesh et al., 2018). Also, IL-12 is the only inflammation marker associated with the transition into psychosis in clinically high risk (CHR) individuals (Goldsmith et al., 2016). However, unlike CHR individuals, IL-1β and IL-10 are elevated in individuals with FEP than controls (Goldsmith et al., 2016). An increased concentration of IL-6 and decline of IL-17 in serum has been revealed in individuals at ultra-high risk (UHR) for psychosis, which explains the association that increased IL-17 levels can improve disease condition (Zeni-Graiff et al., 2016). And the expression of TNF-α has also been found to worsen the negative symptoms (Goldsmith et al., 2019). Collectively individuals with FEP and chronic psychosis has been found elevated cytokine levels, including IFN-γ, IL-1 receptor antagonist (IL-1RA), IL-1β, IL-6, IL-8, IL-10, IL-12, sIL-2R, TGF-β, and TNF-α (Goldsmith et al., 2016). However, among all IL-6 and TNF-α, levels were considerably higher in FEP individuals relative to healthy controls (Fraguas et al., 2019). Unlike FEP individuals, the IFN-γ level was lower in chronic outpatients (Goldsmith et al., 2016). Moreover, a meta-analysis study revealed an elevation in IL-1b, sIL-2R, IL-6, and TNF-α in FEP is independent of antipsychotic treatment (Upthegrove et al., 2014). Further inflammation has also been correlated with suicide. As in psychotic patients with mood disorders, increased serum levels of IL-1β and IL-6 have been detected in blood and autopsy brain samples from suicidality compared with both SCZ non-suicidal patients and healthy controls (Black and Miller, 2015). SCZ patients who committed suicide had an extreme rise in microglial density inside the dorsolateral prefrontal cortex, anterior cingulate cortex, mediodorsal thalamus, along with a course of hippocampal microgliosis (Steiner et al., 2008), which explains an extreme and hazardous level of neuroinflammation can induce suicidality in SCZ.

Furthermore, air pollution has also been reported to induce inflammation associated with SCZ pathogenesis (Horsdal et al., 2019). Interestingly, immune genes, most importantly microglia expressed genes, play a central role in the immune system’s interaction with pollution (Peters et al., 2006; Genc et al., 2012). Exposure to traffic-related air pollution (TRAP) revealed an elevated pro-inflammatory cytokine, IL-6, IL-1ß, CD14, and TNF-α in children, when compared to children living in more eco-friendly areas (Calderón-Garcidueñas et al., 2008, 2015; Gruzieva et al., 2017). Additionally, polluted air evoked an identical inflammatory cytokine profile in a healthy young population, with a characteristic increase of IL-6 and an increased frequency of inflammatory cells and related microparticles, which may indicate endothelial injury (Pope et al., 2016). Moreover, in animal models, TRAP exposure showed an elevated expression of IL-1α, IL-6, and TLR4 in the brain (Bos et al., 2012). TRAP specifically influences the microglial TLR4 signaling in a MyD88-dependent pathway (Woodward et al., 2017). In male children, this interactive inflammatory signaling is subsequently followed by circumstantial and auditory cue fear conditioning and anxiety along with behavioral deficits (Bolton et al., 2012, 2013, 2017). With such a condition, prolonged brings harmful consequences like altered synaptic plasticity (Gruol, 2015) altered brain development and function, featured on MIA models of SCZ (Girgis et al., 2014).

## 5 Resistance and Side Effects of the Drug in NPDs

Treatment of NPDs with the drug has been challenging due to the involvement of diverse etiopathology, where drug resistance is posing a threat to the treatment due to improper counseling during treatment, post-zygotic genetic changes (Rylaarsdam and Guemez-Gamboa, 2019), unpredictable immune response, differential hormonal response, sex-specific (Jacquemont et al., 2014; Desachy et al., 2015) and the dose-dependent response of the drug (McCracken, 2005; Srisawasdi et al., 2017). The interference of drug in metabolic pathways also contribute to unforeseen development of metabolic disorders, and lastly, the drug addiction and behavioral issues in response to minimal treatment time and disease severity ensure difficulties towards drug-associated treatment (Scahill et al., 2016; Srisawasdi et al., 2017; Libowitz and Nurmi, 2021)**.** Current treatment strategies for NDDs include psychotherapy, anti-inflammatory drugs, and antipsychotic drugs with significant side effects.

Meta-analysis studies revealed that nonsteroidal anti-inflammatory drugs hold considerable impact on schizophrenic total, positive and negative symptoms (Sommer et al., 2012). However, this effect is only in subjects with short-term disease or first episode manifestation (Nitta et al., 2013). Moreover, individuals with short-term (0–2 years) disease duration significantly benefited from the same treatment combined with celecoxib. In contrast, patients with longer disease duration and chronic inflammatory diseases did not differ from the placebo control. Anti-inflammatory treatment involving IFN-γ also comes with side effects, including a high risk of unexpected immune responses. Although long-term anti-inflammatory treatment in chronic SCZ has a considerable positive impact this also has been associated with higher inflammation and poorer response to antipsychotics (Grüber et al., 2014; Fond et al., 2020; Hong and Bang, 2020). Exposure to psychological stressors causes alteration and fluctuation in basal cortisol levels (Bradley and Dinan, 2010), correlated with fluctuating psychological symptoms. Antipsychotic medication directly affects cortisol secretion (Cohrs et al., 2006; Cherian et al., 2019). Celecoxib (cyclooxygenase-2 (COX-2) inhibitor) treatment along with risperidone has been significantly more efficient than the patients receiving risperidone alone in SCZ and had a positive effect on cognition (Müller et al., 2006). However, this therapeutic effect is only restricted until the first years of the schizophrenic disease progression (Müller et al., 2010). Risperidone (a second-generation atypical antipsychotic) treatment causes glucose dyshomeostasis and endocrine dysregulation in autistic children and young adults, bringing leptin and insulin resistance in a dose and treatment duration-dependent manner. Moreover, in children and teenagers suffering ASD, termination of medication is associated with aggressive behavior; thus, a long-life medication is suggested (McCracken, 2005; Srisawasdi et al., 2017). Individuals receiving a high dose of risperidone treatment for the long term are at increased risk of developing type 2 diabetes mellitus (Zuo et al., 2013).

Further, administration of olanzapine and aripiprazole for a short duration directly impacts tissue function enhances peripheral insulin resistance, independent of weight gain in healthy people (Teff et al., 2013). A second-generation antipsychotic treatment including amisulpride, clozapine, olanzapine, quetiapine, risperidone has been demonstrated to be associated with increased appetite, and gain in weight may be through interacting with dopamine receptors (Esen-Danaci et al., 2008). Besides, antipsychotic drugs administered to children and adolescents come with a risk of dyslipidemia and expression of HDL-C, and triglycerides are the main lipid biomarkers for cardiometabolic syndrome in SCZ (de Hert et al., 2011). PUFAs such as omega-3 and omega-6 protect the neuronal cells from oxidative damage inflammation, regulate neurogenesis and preserve overall neuronal function (Hashimoto et al., 2014; S. W.; Kim et al., 2016), therefore involved in many mental health conditions and related diseases such as SCZ (Amminger et al., 2015). In SCZ, patients have considerably low levels of omega-3 in the cell membranes (Hoen et al., 2013). Additionally, antipsychotic treatments have been reported to decrease the PUFAs levels (S. W. Kim et al., 2016). Antipsychotics can also potentially intervene in the metabolic process and biomarker levels, including the metabolites from the kynurenic pathway (Condray et al., 2011; Myint et al., 2011).

Twenty-four weeks of treatment with risperidone resulted in a significantly lower abundance of fecal *E. coli*, *Lactobacillus* spp. *Bifidobacterium* spp. when compared with healthy controls, suggesting that risperidone treatment causes a distinctive amendment in fecal microbiota, indicating metabolic changes induced by antipsychotic medication (Yuan et al., 2018). As discussed previously, microbial species have been substantially associated with disease severity, poor cognitive performance, and diagnosis. And antipsychotic treatment can influence the gut microbiota but cannot completely reconstruct the dysbiotic microbiota related to SCZ (Zhu et al., 2020). Current microbiome therapies have shown promising results in managing NDDs and metabolic disorders. Therefore, manipulating gut microbes with enrichment of beneficial microbes to create a balance and other microbiome therapy may have duplex therapeutic potential for neurodevelopmental and metabolic disorders.

## 6 Future Perspective on the Microbiome and Therapeutic Interventions for Neurodevelopmental Psychiatric Disorders

**FIGURE 5:**
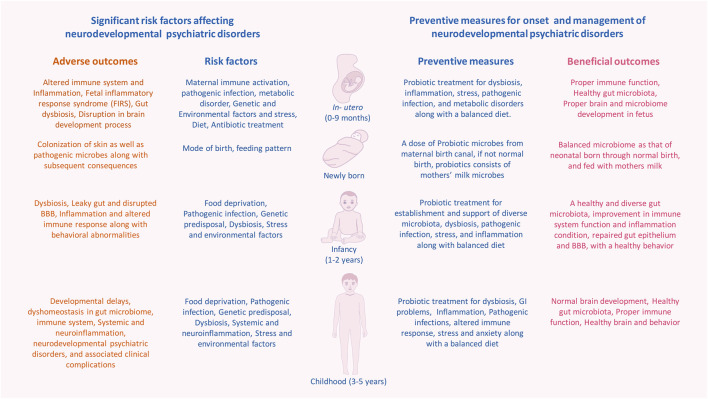
Probiotic therapy to overcome adverse effects NDDs at developmental time points. Probiotic treatment at specific time points such as *in-utero*, after immediate birth, infancy and childhood may help and support the establishment of stable and healthy gut microbiota, prevent the dysregulation in the developmental process, and play the role of a crucial preventive measure for the onset of neurodevelopmental and psychotic disorders *via* neutralizing the effects of inducing factors.

NDDs can be readily linked with gut microbial dysbiosis and inflammatory state, which affect the development of learning, language, cognitive, motor, and behavioral skills with lifelong consequences (Jeste, 2015). Early identification of infants at risk for NDDs is a prerequisite for taking therapeutic measures hypothesized in (Figure 5). There is an imbalance in *Bifidobacteria* sp. *Lactobacillus* sp. and a deficiency of potential SCFA producing microbes in the gut microbiota of individuals suffering from NDD (Bojović et al., 2020). Treatment with the probiotic strain of *Bifidobacteria* sp. and *Lactobacillus* sp. and probiotic bacterial species to normalize the dysbiosis associated with inflammation, neuropsychological distress, GI, and behavioral problems. Moreover, treatment with *B. fragilis* has been reported to reduce the intestinal permeability, normalize the organization of the gut microbiota, and ease disease symptoms in the ASD mice model (Glibert et al., 2013; Hsiao et al., 2013). Administration of probiotics, *Lactobacillus rhamnosus* and *Lactobacillus reuteri*, have improved barrier integrity and function by regulation of the tight junction proteins expression and thereby reducing the microbial translocation *in-vitro* or in animal models (Ulluwishewa et al., 2011; Dicksved et al., 2012). *Lactobacillus* and *Bifidobacteria* were proven to decrease anxiety symptoms considerably (Rao et al., 2009). Supplementation of the probiotic formulation containing *Lactobacillus*, *Bifidobacteria*, and *Streptococci* has been reported to normalize the *Bacteroidetes/Firmicutes* ratio along with imbalanced abundance *Desulfovibrio* sp. and *Bifidobacterium* sp. in the fecal samples of autistic children, as with that of healthy controls (Tomova et al., 2015). In addition to probiotic therapy, fecal microbiota transplantation (FMT) and microbiota transfer therapy (MTT) have been increasingly considered promising therapies in treating NDDs. FMT has been reported to treat IBD and IBS successfully, resulting in a typical gut microbiota composition in patients, improving constipation symptoms (Aroniadis and Brandt, 2013; Rossen et al., 2015). Microbiota transfer therapy (MTT) is a kind of modified FMT. The patient goes through antibiotic treatment for 14 days and then cleans the bowel before administrating a high initial dose of systematized human gut microbiota (SHGM) for up to 7–8 weeks. MTT in a clinical trial has been reported to improve GI symptoms such as indigestion, abdominal pain, constipation, and diarrhea and symptoms related to ASD and normalized any alteration in the gut microbiota in ASD patients (D. W. Kang et al., 2017, 2019). Thus, there is increasing interest in using FMT and MTT as a therapeutic intervention for treating children with NDDs, following all the concerning safety issues (Gaonkar, 2021).

## 7 Conclusion

There is a bidirectional communication network between the CNS and the microbiome ecosystem that we possess (Carabotti et al., 2015). To understand this relationship in a more elaborative way still much has to be discovered. Disturbances in the simultaneous coordinated process of neuronal as well as gut-microbiome development due to overdoses of antibiotics in infants can lead to an inflammatory state at this critical phase of brain development (Neuman et al., 2018). Compositional alterations in the gut microbiome can result in systemic inflammation and neuroinflammation (Belkaid and Hand, 2014). Moreover, the microbiome also plays an essential role in the process of microglial maturation and can modulate glial activation in CNS, which also has been considered as regulating factor of neuroinflammation in the CNS (Abdel-Haq et al., 2019). All these events during the developmental process put up a foundation for the onset of NDDs like ASD, ADHD, SCZ, etc. Investigating intricate pathways for a brief illustration of the microbiome-gut-brain-immune axis in developing NDDs, disease onset, and progression will be beneficial towards the discovery of clinically relevant targeted biotherapies to combat the continuous rise in worldwide NDDs. The future perspective holds the exciting potential of sophisticated microbiome-based therapeutics to prevent the emergence and treatment of NDDs (Figure 5). Further studies are required to elucidate specific molecular signaling pathways associated with processes neuronal development, related disease, diagnosis, and to develop individualized microbiome therapeutics.
